# Instrumented strength assessment in typically developing children and children with a neural or neuromuscular disorder: A reliability, validity and responsiveness study

**DOI:** 10.3389/fphys.2022.855222

**Published:** 2022-10-19

**Authors:** Ineke Verreydt, Ines Vandekerckhove, Elze Stoop, Nicky Peeters, Vanessa van Tittelboom, Patricia Van de Walle, Marleen Van den Hauwe, Nathalie Goemans, Liesbeth De Waele, Anja Van Campenhout, Britta Hanssen, Kaat Desloovere

**Affiliations:** ^1^ Department of Rehabilitation Sciences, KU Leuven, Leuven, Belgium; ^2^ Clinical Motion Analysis Laboratory, University Hospitals Leuven, Pellenberg, Belgium; ^3^ Department of Rehabilitation Sciences, Ghent University, Ghent, Belgium; ^4^ Research Group MOVANT, Department of Rehabilitation Sciences and Physiotherapy (REVAKI), University of Antwerp, Wilrijk, Belgium; ^5^ Multidisciplinary Motor Centre Antwerp (M2OCEAN), University of Antwerp, Antwerpen, Belgium; ^6^ Heder, Laboratory of Clinical Movement Analysis Antwerp, Antwerpen, Belgium; ^7^ Department of Child Neurology, University Hospitals Leuven, Leuven, Belgium; ^8^ Department of Development and Regeneration, KU Leuven, Leuven, Belgium; ^9^ Pediatric Orthopedics, Department of Orthopedics, University Hospitals, Leuven, Leuven, Belgium

**Keywords:** cerebral palsy, Duchenne musclar dystrophy, muscle weakness, instrumented strength assessment, clinimetric properties, reliability, validity, responsiveness

## Abstract

The aim of this study was to determine the clinimetric properties, i.e., reliability, validity and responsiveness of an instrumented strength assessment in typically developing (TD) children and children with cerebral palsy (CP) and Duchenne muscular dystrophy (DMD). Force (N), torque (Nm) and normalized torque (Nm/kg) were defined for maximal voluntary isometric contractions (MVICs) of the lower limb muscles using a pre-established protocol. Intraclass correlation coefficient (ICC), standard error of measurement (SEM) and minimal detectable change (MDC) of TD children (*n* = 14), children with CP (*n* = 11) and DMD (*n* = 11) were used to evaluate intra-rater reliability for the three cohorts and the inter-rater intersession as well as inter-rater intrasession reliability for TD children. Construct validity was assessed by comparing MVICs in TD children (*n* = 28) to children with CP (*n* = 26) and to children with DMD (*n* = 30), using the Kruskal Wallis and post-hoc Mann-Whitney U tests. Responsiveness was investigated by assessing changes in MVICs following a strength intervention in CP (*n* = 26) and a 1 and 2 year follow-up study in DMD (*n* = 13 and *n* = 6, respectively), using the Wilcoxon Signed-Rank test. The overall intra-rater reliability, was classified as good to excellent for 65.1%, moderate for 27.0% and poor for 7.9% of the measures (47.6%, 76.2%, and 66.7% good-excellent; 28.6%, 23.8%, and 33.7% moderate; 23.8%, 0%, and 0% poor in TD, CP, and DMD, respectively), while ICC values for TD children were slightly lower for inter-rater intrasession reliability (38.1% good-excellent, 33.3% moderate and 26.6% poor) and for inter-rater intersession reliability (47.6% good-excellent, 23.8% moderate and 28.6% poor). Children with CP and DMD were significantly weaker than TD children (*p* < 0.001) and the majority of these strength differences exceeded the MDC. Children with CP significantly improved strength after training, with changes that exceeded the SEMs, whereas only limited strength decreases over time were observed in the DMD cohort. In conclusion, the investigated instrumented strength assessment was sufficiently reliable to confirm known-group validity for both cohorts and could detect the responsiveness of children with CP after a strength intervention. However, more research is necessary to determine the responsiveness of this assessment in children with DMD regarding their natural decline.

## 1 Introduction

Muscle weakness is a common symptom in childhood onset disorders like cerebral palsy (CP) and Duchenne muscular dystrophy (DMD), despite its different etiology in both patient groups (Sussman, 2002; Odding et al., 2006). CP is primarily a neurological disease, where the loss of muscle strength is related to neurological factors, namely lower motor drive and altered recruitment patterns. However, secondary impairments caused by the initial brain lesion lead to, among others, altered muscle structure, also contributing to muscle weakness (Hoffman et al., 1988; Sussman, 2002; Mockford and Caulton, 2010). DMD on the other hand, a genetic dystrophy, is classified as a neuromuscular disease, but the origin of the muscle weakness is solely found in the muscular system (Sussman, 2002).

CP is the most common neurological disorder in children, with a prevalence of 2–3 per 1,000 live births (Surveillance of cerebral palsy in Europe: a collaboration of cerebral palsy surveys and registers. Surveillance of Cerebral Palsy in Europe (SCPE)., 2000). Muscle weakness is one of the primary symptoms that is caused by an upper motor neuron lesion occurring in the developing fetal or infant brain (Odding et al., 2006; The Definition and Classification of Cerebral Palsy, 2007). Dallmeijer et al. (2017) described lower limb strength for children with CP in comparison to typically developing (TD) children using handheld dynamometry (HHD) and showed that hip flexion (HF) was most affected, with a reduction of 63%–82%, followed by hip abduction (HA, 47%–76%), knee extension (KE, 56%–68%), knee flexion (KF, 36%–68%) and plantar flexion (PF, 37%–57%) (Dallmeijer et al., 2017).

DMD is a progressive X-linked muscular disease, affecting 2–3 per 10,000 new-born boys (Sussman, 2002). The protein dystrophin, important for muscle cell stability, is deficient due to a mutation in the gene encoding for this protein (Hoffman et al., 1988; Sussman, 2002; Birnkrant et al., 2018). The quick deteriorating muscle dystrophy results in progressive loss of muscle strength and alterations in posture and gait (Sutherland, 1981; Pasternak et al., 1995; Sussman, 2002). First symptoms occur before the age of 5 years, with an early effect on the proximal muscles and eventually resulting in a general muscle impairment (Hoffman et al., 1988; Baumann, 2003). Children with DMD lose ambulation between the age of 7.1 and 18.6 years (mean age: 12.7 years) (Goemans et al., 2021). Mathur et al. (2010) showed that dorsiflexion (DF), PF and KE muscle strength was 67%, 67%, and 71% of TD children, respectively, in boys with DMD (Mathur et al., 2010). Longitudinal analyses in DMD revealed that before the age of 7.5 years KE and KF isometric and isokinetic muscle strength still increased, however to a lesser extent than in TD (Lerario et al., 2012). After the age of 7.5 years, strength in these muscle groups decreased, with a more pronounced decrement after the age of 9 years (Lerario et al., 2012).

While DMD has an intrinsic progressive character, children with CP present with persisting, non-progressive brain damage and variable muscle weakness (Sussman, 2002; The Definition and Classification of Cerebral Palsy, 2007). Consequently, muscle strength training is often included in the treatment protocol of CP, whereas in children with DMD, other modalities, like pharmacological interventions, are considered more useful (Manzur et al., 2008; Scholtes et al., 2008; Vulpen et al., 2017). While several studies suggested the effectiveness of strength training in children with CP (Ryan et al., 2017), valid quantitative measures of muscle strength are considered essential to define the intensity of the strength training program, to monitor adjustments and to properly assess the effects of strength training (Van Vulpen et al., 2013). In children with DMD, the natural history of the disease, including the age at which they lose ambulation, might be altered with promising novel therapeutic strategies. However, demonstrating the benefits of these novel drugs in DMD has shown to be very challenging with the current assessment methods (Goemans et al., 2014). To delineate the natural history of the disease and the potential effect of novel therapies in children with DMD, a reliable and valid assessment method is needed.

A wide range of instruments can be used to assess muscle strength in pediatric populations. While functional testing, such as heel raises and squatting, recently gained popularity, in a clinical setting, the Medical Research Council (MRC) scale is most often used due to its simplicity and all-round applicability. It examines the dynamic strength over the joint range of motion per muscle group of the patient by grading it on an ordinal scale from zero to five. Although this assessment is useful to determine the influence of muscle weakness on a patient’s daily life abilities, it has a subjective character and has questionable inter-rater reliability (Pfister et al., 2018). Isokinetic dynamometry is considered the most valid method to assess muscle strength in adults, due to its dynamic nature, allowing quantification at relevant joint velocity and over relevant joint range of motion (Dallmeijer et al., 2011; El Mhandi and Bethoux, 2013). Yet, this method was found to be challenging in young children or children with distinct muscle weakness and its high costs and large size make it less useful in clinical settings (El Mhandi and Bethoux, 2013). In previous studies, a HHD was often used to assess muscle strength in pediatric populations. In these assessments, participants were asked to perform maximal voluntary isometric contractions (MVICs) against the HHD (Physio and Galea, 2007; Verschuren et al., 2008). Although this method is found to be more reliable than the MRC scale, the strength assessment can be influenced by the assessor (Physio and Galea, 2007; Verschuren et al., 2008; Hébert et al., 2011). Ideally, every assessor should generate the same force while holding the HHD in the test procedure matched to the participant’s force to ensure a true isometric contraction (Physio and Galea, 2007; Hébert et al., 2011). Moreover, when the child is not thoroughly fixated, the obtained results can be influenced by compensation mechanisms (Dallmeijer et al., 2011; Hébert et al., 2011; Goudriaan et al., 2018a). An additional limitation is the static nature of the measurement, limiting its outcome to the unchanged specific joint angle in which strength was assessed, which may not be representative for muscle use in dynamic conditions (Dallmeijer et al., 2011). To eliminate the two other main limitations of HHD, i.e., influence of the strength of the assessor and compensations of the subject, Goudriaan et al. (2018a) developed a new isometric strength assessment protocol. This latter protocol is the main focus of the current study and is further referred to as the ‘instrumented strength assessment’. During this assessment, MVICs were performed in a custom-made chair, i.e., an external frame on which the HHD could be attached. The child was fixated in the chair.

Reliability, validity and responsiveness, which are essential clinimetric properties (Feinstein, 1987; De Vet et al., 2003), were only partly defined for this instrumented strength assessment. The reliability was assessed in TD children for DF, PF, KE, and KF strength, and was found to be moderately reliable in this population, with 13 out of 16 ICCs being higher than 0.500 (Goudriaan et al., 2018a). Construct validity was investigated in both TD and CP children for the ankle and knee joint by associating joint strength with gait parameters. However, clinimetric properties are not randomly transferable from adults to children or from TD children to children with a neural or neuromuscular disorder (De Vet et al., 2003; Jerosch-Herold, 2005; Clark et al., 2017). Associated disabilities such as spasticity and cognitive deficits may influence the test performance of the child (Physio and Galea, 2007). It is therefore important to determine clinimetric properties per specific pediatric clinical population, such as CP and DMD (Clark et al., 2017). Moreover, the responsiveness of this assessment, comparing changes following interventions with reliability indices, are still absent.

The overall aim of the current study was to comprehensively determine the reliability, validity, and responsiveness of the instrumented strength assessment in TD children, as well as in children with CP and DMD in muscle groups around the ankle (DF and PF), knee (KE and KF), and hip [HA, hip extension (HE) and HF]. To achieve this overall aim, the current study was divided into three parts. Part one aimed at determining the intra- and inter-rater reliability of the instrumented strength assessment using the intraclass correlation coefficient (ICC) and the standard error of measurement (SEM) as relative and absolute reliability index, respectively (Goudriaan et al., 2018a). While the intra-rater reliability was assessed in the three cohorts, inter-rater reliability was only assessed in the TD cohort. It was hypothesized that intra-rater reliability of the strength assessment for lower limb muscle groups is good (ICC>0.750) for TD children, as well as for children with CP and DMD, with a tendency of higher reliability in TD children because the performance of muscle strength assessments in children with CP and DMD is expected to be more challenging and thus less consistent (Koo and Li, 2016). Part two aimed to evaluate the construct validity of this instrument through the evaluation of the known-group validity by comparing TD, CP, and DMD cohorts, using the SEM and minimal detectable change (MDC) as a reference value. It was hypothesized that the SEM and MDC values resulting from the reliability analysis are sufficiently small to distinguish strength assessments between pathological and TD populations. Part three aimed to evaluate the responsiveness of the instrumented strength assessment by comparing the SEM and MDC values to change over time during a strength intervention in children with CP and during the natural decline in children with DMD. It was hypothesized that the SEM and MDC values are small enough to define changes over time related to strength training in CP or to the natural evolution in DMD.

## 2 Materials and methods

The methods are described per study part. This study was approved by the ethical commission of KU Leuven (Ethical Committee UZ Leuven/KU Leuven; S59945, S61324, and S63340) and Ghent University (EC/2017/1674) under the Declaration of Helsinki. Participants’ parents/caregivers signed a written informed consent prior to participation. Children above the age of 12 were asked to sign an assent as well.

### 2.1 Part one: reliability of the instrumented strength assessment

#### 2.1.1 Participants

Prior to the study, the sample size was determined based on the approximations of Walter et al. (1998). Corresponding to the ICC-range reported by Goudriaan et al. (2018a), the minimal ICC-value (⍴0) and maximal ICC-value (⍴1) was set at 0.500 (fair to good) and 0.900 (excellent), respectively. Taking into account an alpha of 0.05 and a power of 0.80, a minimal sample size of nine children was necessary in each reliability analysis (inter- and intra-rater) (Walter et al., 1998). To foresee drop-out during the study, the number of participants recruited was larger than the calculated sample size. The final cohorts of 15 TD, 11 CP, and 11 DMD participants were recruited based on predefined inclusion and exclusion criteria, listed in Table 1. For the children with DMD, chronic treatment with corticosteroids and participation in clinical trials were permitted.

**TABLE 1:** Overview of the in- and exclusion criteria for the reliability analysis.

	Inclusion criteria	Exclusion criteria
TD	- Age between five and 18 years old	- History of orthopedic impairments of the lower limb
		- Neurological disorders
		- Known cognitive or behavioral disorders
CP	- Age between five and 18 years old	- Less than 6 months post-BTX
	- Confirmed diagnosis of spastic CP	- Less than 12 months post-surgery
	- GMFCS I-III	- Inability to understand the test procedure
DMD	- Age between five and 18 years old	- Cognitive and behavioral disorders preventing accurate measurements
	- Diagnosis of DMD *via* immune-histochemistry, muscle biopsy and/or mutation of the dystrophin gene	- History of lower limb surgery
	- Ambulant and able to walk independently for at least 100 m	- Clinical picture of Becker muscular dystrophy
		- Genetic diagnosis predicting a milder phenotype, such as in frame deletions

Abbreviations in alphabetical order: BTX, botulinum toxin infiltration; CP, cerebral palsy; DMD, Duchenne muscular dystrophy; GMFCS, gross motor function classification system; m, meter; TD, typically developing.

#### 2.1.2 Study design

**FIGURE 1:**
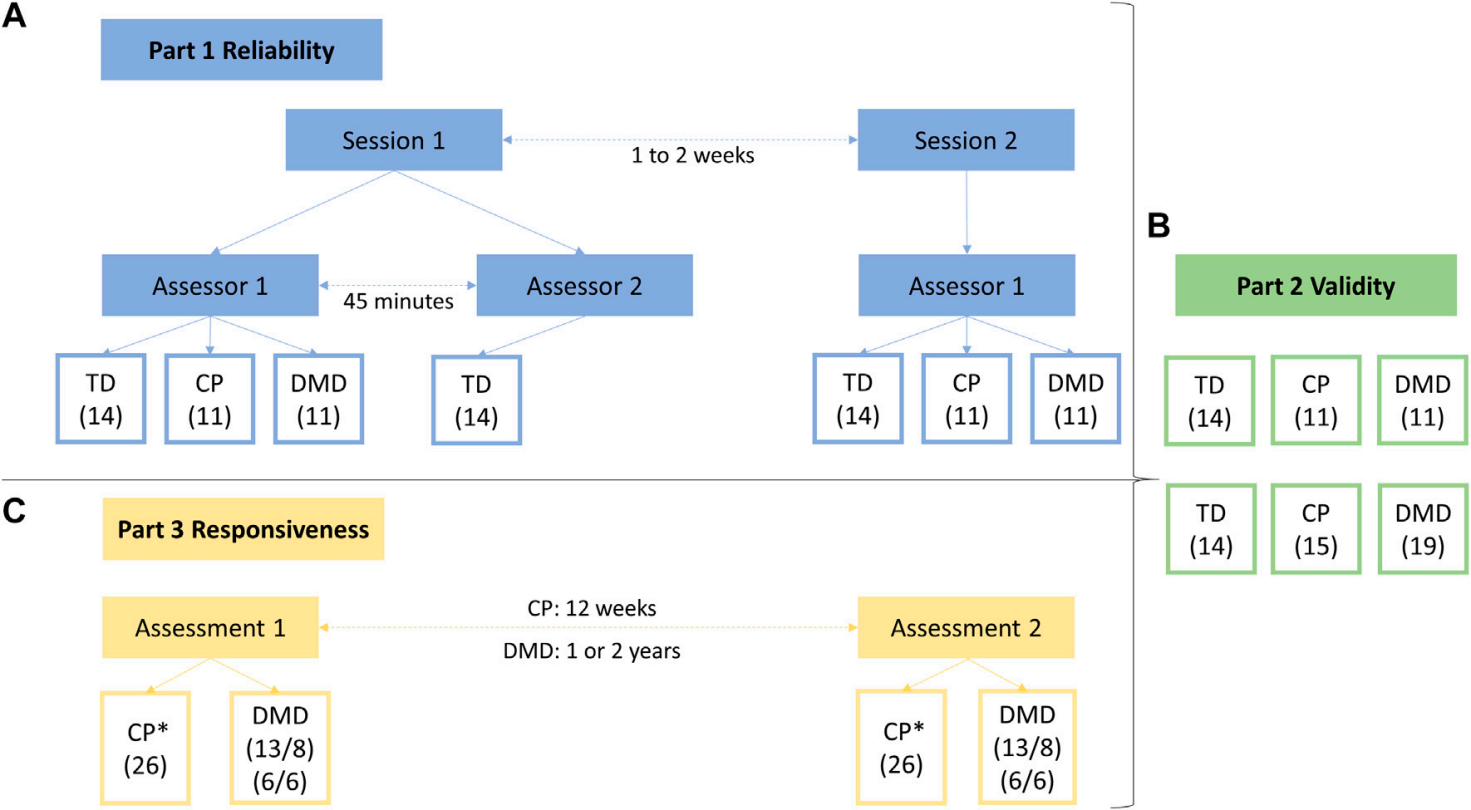
Study design of all three parts of the study. Part one represents the reliability study **(A)**, including (1) the intra-rater reliability, with data from session 1—assessor 1 and from session 2—assessor 1, (2) the inter-rater intrasession reliability, with data from session 1—assessor 1 and from session 1—assessor 2 and (3) the inter-rater intersession reliability, with data from session 1—assessor 2 and from session 2—assessor 1. Part two represents the validity study **(B)**, based on the data of the children included in the reliability study complemented with data of additional participants. Part three represents the responsiveness study **(C)**, based on (1) data of a strength training program in children with CP (* indicating that the hip joint was not assessed) and (2) data of the natural decline of the children with DMD. Abbreviations in alphabetic order: CP, cerebral palsy; DMD, Duchenne muscular dystrophy; TD, typically developing.

First, the intra-rater reliability of DF, PF, KE, KF, HA, HE, and HF MVICs was investigated for the three cohorts, TD, DMD, and CP, and was defined by measuring all children twice by the same assessor with an interval of 1–2 weeks. An interval of 1–2 weeks was set to avoid the influence of recall and to avoid further deterioration of muscle weakness in the DMD population and progression of the clinical picture in the CP population. Second, the inter-rater reliability in an intrasession condition was defined for the same muscle groups by performing a second measurement by another assessor 45 min after the first measurement, on the first test day. Third, the inter-rater intersession reliability was defined by comparing this second measurement performed on the first test day with the data collected during the second session. The inter-rater reliability, both intersession and intrasession, was limited to the TD cohort to reduce the test load for the pathological cohorts. Assessors were a well-trained senior human movement scientist and pediatric physiotherapist, and two final year master students in pediatric physical therapy who received training to perform the instrumented strength assessment (1 day of general explanation, 2 days of practice and assisted with 10 measurements). After this initial training, the students collected the inter-rater and intra-rater reliability assessments in the TD cohort, whereby the data collection during the first and second assessments on the first test day was supervised by the human movement scientist or pediatric physiotherapist, while the third assessment during the second session was collected without supervision. All assessments for the intra-rater reliability in the CP and DMD cohort were collected by the senior human movement scientist or pediatric physiotherapist. Figure 1A shows an overview of the study design. Data was collected unilaterally for all children. The side was randomly selected for the TD children and the most affected side was selected for the children with CP and DMD, while the evaluated limb was randomly selected in case of a symmetrical clinical picture. In all cohorts, the same limb was evaluated in each measurement session.

#### 2.1.3 Data collection

**FIGURE 2:**
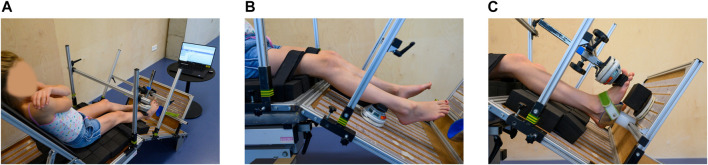
Custom-made chair used for assessment of maximal voluntary isometric contractions. The chair and handheld dynamometer are positioned to assess knee extension strength **(A)**. Further, the whole measurement set-up is shown with the laptop placed in front of the child to give visual feedback. Close-up on the position of the handheld dynamometer for knee flexion strength **(B)**. Close-up on the position of the handheld dynamometer for dorsiflexion strength **(C)**.

Anthropometric data, including height and weight, was obtained during the first measurement session. Muscle strength was defined by performing MVICs using a HHD (MicroFet, Hogan Health Industries, West Jordan, UT United States) in a standardized manner, as introduced by Goudriaan et al. (2018a). Thereby, a custom-made chair was used, fixating the hip joint in 60° flexion, the knee joint in 30° flexion and the ankle joint in a neutral position. A visual impression of the measurement set-up is given in Figure 2. When positioned in the chair, segment lengths of the lower limb (hip: trochanter major—knee joint space; knee: proximal border of fibula head—distal border of lateral malleolus; ankle: distal border (dorsal point) of lateral malleolus—distal metatarsal II, projected on the lateral border of the foot) were measured. The HHD was placed at 75% of the segment length to standardize the lever arms. At each measurement session, the segment lengths and lever arms were determined by the assessor performing the measurement. Compensations were minimized by fixating the child in the chair using a waistbelt, two thigh straps and performing the MVICs crossing their arms in front of their chest. During the assessments of DF, PF, HE, and HF, the heel was fixated in a heel cuff. Influence of gravitational force during the MVICs of PF, KF, and HE was ruled out by performing a separate passive trial and subtracting it from the actual MVICs outcomes (Boiteau et al., 1995). The children were asked to perform a test trial, followed by three well executed actual trials with a duration of 3–5 s. If compensations were observed, i.e. obvious contractions in other muscles than the tested muscle, an additional trial was performed after verbal instructions to correctly perform the MVIC and avoid compensations. Between each trial, a resting period of at least 10 s was provided. In case of observed signs of fatigue, the recovery period was prolonged until the participant was ready. When transitioning to the measurements of a different joint, a resting period of at least 30 s was provided. The children received both consistent verbal encouragement by the assessor and visual feedback of the ongoing trial as well as previous trials of that assessment.

#### 2.1.4 Data analysis

All data was analyzed using a custom-written Matlab (The Mathworks Inc., Natick, M.A., R2019a) script. At first, the strength data was resampled to 100 Hz. The maximal force (in Newton [N]) per MVIC trial was extracted. The average of the maximal force from the representative MVICs was calculated for each muscle group. By multiplying the average maximal force with the lever arm and dividing it by the body weight, the mean torque (in Newton meter [Nm]) and mean torque normalized to body weight (in Newton meter per kilogram body weight [Nm/kg]) were calculated, respectively. Hereby, torque and normalized torque were considered the primary outcomes. The force was described as secondary outcome, as it may help to understand the observed torque values.

#### 2.1.5 Statistical analysis

To determine the inter- and intra-rater reliability, the ICCs for MVICs were calculated in SPSS (SPSS Inc., Chicago, IL version 27). ICC (2,1) with a 95% confidence interval (CI) was calculated using a two-way random model based on a single-rater with absolute-agreement. Bland-Altman plots were created and checked to determine any systematic bias. Based on visual inspection of these plots, two assessors independently checked for outliers, i.e., a participant from which the difference between the two assessments exceeded 2 standard deviations. In case outliers were caused by a processing error, the MVICs were reprocessed. In addition, one TD participant (all MVICs), the PF MVICs of one participant with CP and the HA MVICs of one participant with DMD were outliers and were excluded for further analysis because of the following reasons: an exceptional more advanced maturity compared to other participants, a compensation during the assessment that was discovered by a deviating selectivity score [assessed during standard clinical examination with the Selective Control Assessment of the Lower Extremity (Fowler et al., 2009)] and missing data of the first assessment, respectively. Following Koo and Li (2016), an ICC ≤ 0.500 resembled poor reliability, 0.501–0.750 a moderate, 0.751–0.900 a good and >0.900 an excellent reliability (Koo and Li, 2016). The SEM was calculated by taking the square root of the mean square error, which is the within group mean square value in the two-way random model. If the SEM is low, the reliability is high, which is associated with an ICC approaching one. In addition, the MDC score was calculated using the SEM obtained in the two-way ANOVA by SEM*1.96*√2. Both SEM and MDC were also expressed as a percentage of the median of the averaged scores from each assessment per participant, SEM% and MDC%, respectively.

### 2.2 Part two: validity of the instrumented strength assessment

#### 2.2.1 Participants

The required sample size was estimated based on previous research (Goudriaan et al., 2018a; Vandekerckhove et al., 2020). The Wilcoxon effect sizes r (Fritz et al., 2012) of the differences in muscle strength between CP and TD were calculated from the available data of Goudriaan et al. (2018a) and ranged between 0.55 and 0.80 (Goudriaan et al., 2018a). Vandekerckhove et al. (2020) reported similar Wilcoxon effect sizes r ranging between 0.65 and 0.79 for differences in muscle strength between DMD and TD (Vandekerckhove et al., 2020). Taking into account a Cohen’s D effect size of 1.3, which corresponds to the smallest Wilcoxon effect size r (0.55) reported in previous studies, an α error probability of 0.0036 (to correct for the comparison of 14 parameters) and a power of 0.95, a minimal sample size of 26 for each cohort was required. Therefore, the children from the reliability study were supplemented with 14 additional TD children, 15 additional children with CP and 19 additional children with DMD. These additional data were collected as part of ongoing studies, i.e., a natural history in children with DMD, and databases within a larger project for the TD and CP cohort. For the TD cohort, the assessors of these additional data were trained final year master students in pediatric physical therapy, and for the CP and DMD cohort the assessors were the well-trained senior human movement scientist and pediatric physiotherapist. In total, the TD, CP and DMD cohort included 28, 26, and 30 children, respectively. The same inclusion and exclusion criteria were applied as specified in part one (Table 1).

#### 2.2.2 Study design

Part two of the study investigated the construct validity of the instrumented strength assessment for MVICs for all measured muscle groups including all three cohorts, i.e., TD, CP, and DMD. First, the validity was assessed by investigating differences between the TD children and the two clinical cohorts, using unpaired comparison analysis. Second, differences between the median data of TD and CP and between the median data of TD and DMD were compared with the SEM and MDC values of the CP and DMD cohort, respectively. These SEM and MDC values were obtained in part one of the study. Figure 1B shows an overview of the study design.

#### 2.2.3 Data collection

Idem part one.

#### 2.2.4 Data analysis

Idem part one.

#### 2.2.5 Statistical analysis

First, since the data was not normally distributed, the non-parametric Kruskal Wallis test was used to compare the three different cohorts, indicating whether a difference between the TD, CP, and DMD cohort was found. In case of significant results in the Kruskal Wallis test, a post-hoc Mann-Whitney U (MWU) test was conducted to locate these differences. To correct for the comparison of 14 primary parameters, i.e., torque and normalized torque of seven muscle groups, the significance threshold was set to α = 0.0036, according to the Bonferroni correction (Sidak, 1967). Critical *p*-values ranging between 0.0036 and 0.05 were discussed as tendencies. Second, the absolute differences between the median data of TD and CP and between the median data of TD and DMD were calculated for torque and normalized torque, as well as for force, per muscle group. To explore the relevance of these differences, we compared the absolute differences to the absolute SEM and MDC values of the CP and DMD cohort. This comparison was visualized, in the absence of additional statistical testing, and indicated the ability of the instrumented strength assessment to distinguish between a TD cohort and clinical cohorts. To be able to interpret the measurement error in relation to the extent of weakness, the relative differences between the median data of TD and CP and between the median data of TD and DMD (i.e., the absolute difference relative to the median TD scores) were also compared with the SEM% and MDC% values of the CP and DMD cohort, respectively.

### 2.3 Part three: responsiveness of the instrumented strength assessment

#### 2.3.1 Participants

In part three of the study, only children with CP and DMD who participated in ongoing follow-up studies, were included. Due to the lack of previously reported follow-up studies using the same instrumented strength assessment and the explorative nature of our ongoing studies, the required sample size could not be calculated *a priori*. Hence, all available data from the ongoing studies were checked to determine if they could be included in part three of the current study. The assessors of these data were the well-trained senior human movement scientist and pediatric physiotherapist for the CP and DMD cohort. The inclusion and exclusion criteria of these subjects were the same as in part one of this study (Table 1). A maximum age of 12 years was an additional inclusion criterion for the children with CP (defined by the design of the ongoing study). This resulted in a total of 26 children with CP. Even though DMD is a progressive disorder, natural history studies have shown that children with DMD present maturational improvements before the age of 7 years, followed by a period of stability, and finally entering in a more rapid decline (Mcdonald et al., 2013b; Goemans et al., 2016; Jumah et al., 2019). In addition, increases in muscle strength before the age of 7.5 years have previously been reported (Lerario et al., 2012). Therefore, two additional inclusion criteria were used for children with DMD: 1) age > 7.5 years old and 2) an observed motor decline indicated by a decrease in 6 min walking test (6 MWT) > 8% of the 6 MWT at the first measurement, which corresponds to the minimal clinically important difference previously reported (Mcdonald et al., 2013a). This way, we ensured that the included DMD patients were in “decline.” Two DMD groups, with a follow-up interval of 1 year and 2 years, were created. The first group (1-year interval) consisted of 13 pairs of measurements from eight children with DMD. Six of these 13 children with DMD were included in the second group (2-year interval).

#### 2.3.2 Study design

The responsiveness of the instrumented strength assessment was defined by investigating differences in MVICs between two measurement sessions, within the CP and DMD cohort. The enrolled children with CP were involved in a strength intervention study that consisted of a 12-week, partially home-based, intervention for the lower limb muscles acting around the ankle and knee. Hence, the hip joint was not included for the responsiveness assessment in children with CP. The strength intervention followed the guidelines of progressive resistance training, prescribing three to four training sessions per week (Verschuren et al., 2011), of which one to three sessions were performed under the supervision of the physiotherapist and the remaining ones at home. The training program consisted of one to three multi-joint exercises, followed by two to three single-joint exercises targeting KE, KF, and PF. Exercises were performed in three sets of ten repetitions, to match an estimated effort of 60%–80% of the 1-repetition maximum, and were gradually progressed. The instrumented strength assessment was performed at baseline and at the end of the intervention. The enrolled children with DMD were involved in a follow-up study that described the natural decline of muscle strength of the ankle, knee and hip muscles over time. Data after 1 year and after 2 years were analyzed. Figure 1C shows an overview of the study design.

#### 2.3.3 Data collection

Idem part one.

#### 2.3.4 Data analysis

Idem part one.

#### 2.3.5 Statistical analysis.

First, Wilcoxon Signed-Rank tests were performed to investigate differences in MVICs between two measurement sessions, i.e., to evaluate whether muscle strength increased in children with CP and decreased in the boys with DMD. The significant threshold was set to 0.0063, to correct for the comparison of eight parameters, in the CP cohort. In the DMD cohort, the significant threshold was set to 0.0036, to correct for the comparison of 14 parameters. *p*-values ranging between the significant thresholds and 0.05 are described as trends. Second, the absolute difference between the MVICs of the two measurements per participant was calculated. The median of all absolute differences was compared with the absolute SEMs and MDCs determined in part one. This comparison was visualized, in the absence of additional statistical testing, and indicated the ability of the instrumented strength assessment to detect the responsiveness in the CP and DMD cohort. The relative difference was calculated as absolute difference between the MVICs of two measurements relative to the MVIC of the first measurement per participant. The median of all relative differences was compared to the SEM% and MDC% from part one.

## 3 Results

In the results section, only the primary parameters are described. The results on the secondary parameters can be found in the corresponding tables.

### 3.1 Part one: reliability

Subject characteristics and median values for the MVICs for the three cohorts of the reliability study are presented in Tables 2 and 3.

**TABLE 2:** Intra-rater intersession reliability results of the TD children as well as children with CP and DMD included in the reliability study.

	TD: Intra-rater intersession reliability (A1-A3)				CP: Intra-rater intersession reliability (A1-A2)				DMD: Intra-rater intersession reliability (A1-A2)			
	Median (IQR)				Median (IQR)				Median (IQR)			
Number	14				11				11			
Age (years)	10.5 (4.0)				15.2 (4.4)				12.2 (2.2)			
Weight (kg)	34.0 (14.5)				51.3 (15.0)				42.8 (14.7)			
Height (cm)	141.5 (15.0)				151.0 (24.7)				128.9 (6.6)			
GMFCS	—				I: 1 II: 6 III:4				—			

	Median (IQR)	ICC (95%CI)	SEM	MDC	Median (IQR)	ICC (95%CI)	SEM	MDC	Median (IQR)	ICC (95%CI)	SEM	MDC
Primary parameter: Torque (Nm)												
Dorsiflexion	10.7 (3.2)	0.914 (0.760–0.971)	1.8	4.9	3.5 (2.5)	0.840 (0.504–0.955)	0.9	2.4	3.7 (3.1)	0.745 (0.307–0.924)	0.8	2.3
Plantar flexion	17.5 (7.9)	0.626 (0.194–0.860)	3.7	10.4	6.6 (4.3)	0.743 (0.193–0.936)	2.0	5.6	8.6 (5.0)	0.743 (0.277–0.924)	1.8	4.9
Knee extension	43.2 (21.8)	0.874 (0.657–0.957)	8.7	24.1	13.4 (18.3)	0.935 (0.779–0.982)	3.0	8.3	15.2 (10.8)	0.915 (0.728–0.976)	2.7	7.4
Knee flexion	27.9 (19.2)	0.911 (0.751–0.971)	3.5	9.6	16 (16.8)	0.719 (0.276–0.914)	5.6	15.6	14.6 (7.3)	0.833 (0.508–0.952)	1.7	4.8
Hip abduction	33.9 (18.8)	0.892 (0.704–0.982)	4.7	13.1	9.4 (4.3)	0.860 (0.582–0.960)	3.2	9.0	16.4 (7.0)	0.728 (0.201–0.926)	2.8	7.7
Hip extension	38.2 (25.3)	0.768 (0.420–0.919)	9.1	25.2	22.7 (31.3)	0.947 (0.795–0.986)	3.6	10.0	20.7 (18.1)	0.863 (0.576–0.960)	3.6	10.1
Hip flexion	58.2 (43.0)	0.622 (0.180–0.859)	17.1	47.3	31.2 (24.7)	0.870 (0.585–0.963)	7.6	21.0	30.5 (15.2)	0.797 (0.352–0.943)	4.1	11.4

Primary parameter: Normalized torque (Nm/kg)												
Dorsiflexion	0.29 (0.05)	0.750 (0.400–0.911)	0.03	0.09	0.08 (0.08)	0.790 (0.414–0.938)	0.03	0.09	0.11 (0.08)	0.760 (0.354–0.928)	0.03	0.09
Plantar flexion	0.44 (0.25)	0.713 (0.333–0.896)	0.10	0.29	0.14 (0.16)	0.797 (0.337–0.950)	0.05	0.15	0.24 (0.19)	0.804 (0.414–0.944)	0.05	0.15
Knee extension	1.30 (0.39)	0.462 (−0.046–0.786)	0.23	0.65	0.3 (0.38)	0.931 (0.761–0.981)	0.05	0.15	0.41 (0.28)	0.905 (0.699–0.973)	0.08	0.21
Knee flexion	0.81 (0.24)	0.862 (0.621–0.954)	0.08	0.21	0.35 (0.42)	0.562 (-0.059–0.862)	0.19	0.52	0.38 (0.19)	0.889 (0.657–0.968)	0.04	0.12
Hip abduction	0.96 (0.22)	0.343 (-0.220–0.730)	0.14	0.40	0.25 (0.24)	0.898 (0.680–0.971)	0.05	0.15	0.44 (0.2)	0.750 (0.262–0.932)	0.08	0.23
Hip extension	1.11 (0.31)	0.121 (−0.429–0.600)	0.22	0.60	0.45 (0.5)	0.946 (0.781–0.986)	0.06	0.18	0.54 (0.46)	0.857 (0.563–0.959)	0.12	0.33
Hip flexion	1.75 (0.38)	0.088 (−0.419–0.568)	0.40	1.12	0.86 (0.71)	0.884 (0.627–0.967)	0.15	0.41	0.81 (0.33)	0.816 (0.434–0.947)	0.12	0.34

Secondary parameter: Force (N)												
Dorsiflexion	104.9 (36.4)	0.922 (0.774–0.974)	13.6	37.7	35.7 (26.1)	0.787 (0.378–0.938)	9.3	25.7	47.8 (35.4)	0.674 (0.199–0.899)	10.6	29.5
Plantar flexion	171.5 (90.3)	0.652 (0.202–0.874)	38.7	107.4	71.7 (46.1)	0.721 (0.149–0.930)	21.2	58.9	103.6 (82.6)	0.775 (0.360–0.934)	23.2	64.3
Knee extension	177.5 (58.7)	0.779 (0.445–0.923)	34.7	96.1	59.5 (59.4)	0.919 (0.729–0.978)	11.0	30.4	79.5 (68.1)	0.909 (0.712–0.974)	13.6	37.8
Knee flexion	116.8 (56.1)	0.890 (0.690–0.963)	12.2	33.8	69.8 (53.2)	0.621 (0.110–0.879)	22.2	61.6	69.2 (38.9)	0.844 (0.531–0.955)	9.4	26.0
Hip abduction	126.4 (38.7)	0.785 (0.446–0.926)	18.5	51.2	35.6 (13.1)	0.846 (0.549–0.956)	10.7	29.7	66.5 (24.1)	0.750 (0.246–0.932)	12.0	33.2
Hip extension	143.5 (43.3)	0.585 (0.124–0.843)	33.7	93.3	90.9 (98.3)	0.939 (0.770–0.984)	12.3	34.2	97.9 (74.9)	0.865 (0.583–0.961)	15.3	42.4
Hip flexion	229.9 (125.1)	0.453 (−0.027–0.778)	54.6	151.4	128.5 (69.4)	0.838 (0.505–0.954)	26.5	73.3	134.7 (44.7)	0.749 (0.233–0.929)	16.9	46.9

Abbreviations in alphabetical order: A1, assessment one; A2, assessment two; A3, assessment three; CI, confidence interval; cm, centimeter; CP, cerebral palsy; DMD, Duchenne muscular dystrophy; GMFCS, gross motor function classification system; ICC, intraclass correlation coefficient; IQR, interquartile range; kg, kilogram; MDC, minimal detectable change; N, Newton; Nm, Newton meters; Nm/kg, Newton meters per kilogram; SEM, standard error of measurement; TD, typical developing; Green, excellent to good reliability; Blue, moderate reliability; Red, poor reliability.

**TABLE 3:** Inter-rater intrasession and intersession reliability results for the TD children included in the reliability study.

	TD: Inter-rater intrasession reliability (A1-A2)				TD: Inter-rater intersession reliability (A2-A3)			
	Frequency or median (IQR)				Frequency or median (IQR)			
Number	14				14			
Age (years)	10.5 (4.0)				10.5 (4.0)			
Weight (kg)	34.0 (14.5)				34.0 (14.5)			
Height (cm)	141.5 (15.0)				141.5 (15.0)			

	Median (IQR)	ICC (95%CI)	SEM	MDC	Median (IQR)	ICC (95%CI)	SEM	MDC
Primary parameter: Torque (Nm)								
Dorsiflexion	10.1 (4.7)	0.936 (0.817–0.979)	1.4	4.0	9.9 (4.3)	0.925 (0.761–0.976)	1.5	4.2
Plantar flexion	14.3 (7.6)	0.234 (-0.365–0.676)	6.2	17.2	16.4 (7.2)	0.397 (-0.122–0.753)	5.3	14.8
Knee extension	40.1 (24.4)	0.936 (0.536–0.984)	4.4	12.3	38.3 (16.5)	0.867 (0.646–0.955)	9.1	25.2
Knee flexion	27.8 (18.7)	0.877 (0.660–0.959)	4.9	13.5	27.1 (16.4)	0.791 (0.469–0.928)	5.9	16.4
Hip abduction	33.6 (17.5)	0.900 (0.723–0.967)	4.8	13.4	32.3 (20.3)	0.874 (0.662–0.957)	5.4	14.9
Hip extension	42.3 (23.7)	0.735 (0.344–0.907)	10.6	29.5	38.0 (20.0)	0.863 (0.628–0.954)	7.9	22.0
Hip flexion	57.7 (36.1)	0.714 (0.297–0.900)	12.9	35.7	56.7 (40.2)	0.787 (0.465–0.926)	10.9	30.1

Primary parameter: Normalized torque (Nm/kg)								
Dorsiflexion	0.26 (0.08)	0.758 (0.398–0.916)	0.03	0.09	0.29 (0.09)	0.757 (0.398–0.915)	0.03	0.09
Plantar flexion	0.44 (0.23)	0.296 (-0.287–0.708)	0.14	0.39	0.47 (0.23)	0.332 (-0.157–0.712)	0.14	0.38
Knee extension	1.11 (0.45)	0.417 (-0.054–0.756)	0.30	0.84	1.18 (0.45)	0.389 (-0.098–0.744)	0.30	0.85
Knee flexion	0.78 (0.31)	0.659 (0.204–0.878)	0.14	0.38	0.78 (0.22)	0.622 (0.140–0.862)	0.14	0.40
Hip abduction	0.91 (0.35)	0.701 (0.307–0.892)	0.12	0.33	0.92 (0.22)	0.502 (0.018–0.803)	0.16	0.45
Hip extension	1.17 (0.34)	0.222 (-0.366–0.668)	0.25	0.71	1.07 (0.31)	0.360 (-0.220–0.742)	0.22	0.61
Hip flexion	1.67 (0.36)	0.209 (-0.226–0.624)	0.33	0.92	1.61 (0.45)	0.344 (-0.219–0.731)	0.30	0.82

Secondary parameter: Force (N)								
Dorsiflexion	101.4 (42.5)	0.899 (0.723–0.966)	13.7	38.1	97.7 (42.1)	0.674 (0.199–0.899)	14.8	41.1
Plantar flexion	147.6 (80.8)	0.171 (-0.411–0.637)	57.9	160.5	162.6 (73.9)	0.214 (-0.330–0.655)	50.7	140.5
Knee extension	174.4 (91.3)	0.903 (0.090–0.979)	12.9	35.8	164.6 (52.6)	0.832 (0.556–0.943)	29.6	81.7
Knee flexion	111.1 (63.5)	0.750 (0.375–0.913)	21.3	59.0	115.2 (62.8)	0.678 (0.239–0.885)	23.5	65.1
Hip abduction	125.4 (56.6)	0.849 (0.604–0.948)	16.8	46.5	118.5 (39.8)	0.782 (0.455–0.924)	21.1	58.6
Hip extension	156.0 (52.6)	0.645 (0.185–0.871)	35.9	99.5	138.1 (54.3)	0.773 (0.422–0.920)	28.0	77.6
Hip flexion	221.2 (100.5)	0.538 (0.055–0.822)	42.6	118.0	219.4 (91.6)	0.694 (0.270–0.891)	35.3	97.8

Abbreviations in alphabetical order: A1, assessment one; A2, assessment two; A3, assessment three; CI, confidence interval; cm, centimeter; ICC, intraclass correlation coefficient; IQR, interquartile range; kg, kilogram; MDC, minimal detectable change; N, newton; Nm, newton meters; Nm/kg, newton meters per kilogram; SEM, standard error of measurement; TD, typically developing; Green, excellent to good reliability; Blue, moderate reliability; Red, poor reliability.

Table 2 shows the ICC, SEM and MDC values of the intra-rater reliability, while the SEM% and MDC% are provided in Supplementary Table S1. Taking the results of the three cohorts into account, 65.1% of the parameters were classified as good to excellent, 27.0% as moderate and 7.9% as poor reliability. In the TD cohort, 47.6% of the parameters were classified as good to excellent, 28.6% as moderate and 23.8% as poor reliability. The ICCs for torque were all classified as good to excellent, except for PF and HF, which were classified as moderate. Normalized torques indicated the lowest reliability, whereby all ICCs around the hip were classified as poor, while ICCs around the knee and ankle were moderate to good, except for KE which showed a poor reliability. In the CP cohort, 76.2% of the parameters were classified as good to excellent and 23.8% as moderate reliability. For torque, ICCs were all classified as good to excellent, except for PF and KF, which were classified as moderate. The ICCs of the normalized torques were all classified as good to excellent, except for KF, which showed a moderate ICC. In the DMD cohort, 66.7% of the parameters were classified as good to excellent and 33.7% as moderate reliability. For torque, ICCs were all classified as good to excellent, except for DF, PF, and HA, which were classified as moderate. All the ICCs for normalized torque for boys with DMD were good to excellent, except for HA, which showed a moderate ICC.

For the TD cohort, the ICC, SEM, and MDC of the inter-rater intrasession and intra-rater intersession are presented in Table 3, while the SEM% and MDC% are provided in Supplementary Table S1. For the inter-rater intrasession, 38.1% of the ICCs were classified as good to excellent, 33.3% as moderate and 28.6% as poor. For torque, the reliability of all muscle groups was classified as good to excellent, except for HE and HF, which were classified as moderate and PF, which was classified as poor. Concerning the ICCs for normalized torques, only for DF, the reliability was classified as good, whereas KF and HA had moderate and PF, KE, HF, and HE had poor reliability. For the inter-rater intersession reliability, 47.6% of the ICCs were classified as good to excellent, 23.8% as moderate and 28.6% as poor. For torque, the ICCs of all muscle groups were classified as good to excellent reliability, except for PF which was classified as poor. Concerning the ICCs for normalized torques, only DF had good reliability, whereas KF and HA had moderate and PF, KE, HF, and HE had poor reliability.

### 3.2 Part two: validity

The descriptive results of the three cohorts included in the validity study and the median values of the MVICs for the three cohorts are presented in Table 4. The children with CP and DMD were significantly weaker than the TD children for all muscle groups (*p* ≤ 0.001), whereas muscle-specific differences were observed between CP and DMD. The children with CP were significantly weaker than the children with DMD in the ankle joint for normalized torque, and for HA normalized torque (*p* ≤ 0.002), whereas children with DMD were significantly weaker than the children with CP for HE torque and normalized torque (*p* < 0.001).

**TABLE 4:** Subject characteristics and median torque, normalized torque and force of the TD, CP, and DMD cohorts and statistical results of the comparison of the three cohorts, included in the validity study.

	TD	CP	DMD	Kruskal–wallis test
Subject information	Median (IQR)	Median (IQR)	Median (IQR)	*p*-value
Number of participants	28	26	30	
Age (years)	10.9 (2.8)	12.0 (4.0)	10.6 (4.2)	*p* = 0.057
Weight (kg)	36.2 (11.8)	42.8 (26.1)	31.9 (20.6)	*p* = 0.136
Height (cm)	144.7 (15.0)	144.5 (18.3)°	125.4 (17.9)*	*p* < 0.001
GMFCS-level	—	I:2 II:16 III:8	—	

Primary parameter: Torque (Nm)				
Dorsiflexion	10.1 (4.6)	1.8 (2.4)*	3.7 (2.4)*	*p* < 0.001
Plantar flexion	16.2 (7.9)	4.7 (5.0)*	9.5 (6.7)*	*p* < 0.001
Knee extension	38.1 (25.1)	12.2 (10.8)*	16.5 (10.9)*	*p* < 0.001
Knee flexion	29.6 (17.1)	11.8 (9.0)*	9.3 (7.8)*	*p* < 0.001
Hip abduction	26.5 (20.2)	8.5 (7.2)*	11.8 (9.8)*	*p* < 0.001
Hip extension	38.5 (18.2)	17.2 (9.1)*°	7.6 (7.3)*	*p* < 0.001
Hip flexion	51.4 (27.3)	25.9 (14.7)*	26.7 (11.0)*	*p* < 0.001

Primary parameter: Normalized torque (Nm/kg)				
Dorsiflexion	0.33 (0.21)	0.05 (0.05)*°	0.12 (0.08)*	*p* < 0.001
Plantar flexion	0.52 (0.47)	0.15 (0.19)*°	0.27 (0.27)*	*p* < 0.001
Knee extension	1.18 (0.44)	0.37 (0.39)*	0.50 (0.32)*	*p* < 0.001
Knee flexion	0.89 (0.33	0.30 (0.26)*	0.33 (0.26)*	*p* < 0.001
Hip abduction	0.90 (0.34)	0.20 (0.21)*°	0.44 (0.27)*	*p* < 0.001
Hip extension	1.25 (0.54)	0.39 (0.36)*°	0.24 (0.32)*	*p* < 0.001
Hip flexion	1.43 (0.64)	0.65 (0.56)*	0.90 (0.53)*	*p* < 0.001

Secondary parameter: Force (N)				
Dorsiflexion	106.3 (47.7)	20.9 (23.7)*°	42.9 (35.1)*	*p* < 0.001
Plantar flexion	162.0 (88.9)	54.0 (46.0)*°	115.1 (81.3)*	*p* < 0.001
Knee extension	162.4 (74.8)	53.4 (51.1)*	82.3 (48.3)*	*p* < 0.001
Knee flexion	121.8 (67.2)	47.9 (26.5)*	49.0 (34.0)*	*p* < 0.001
Hip abduction	110.5 (52.0)	33.1 (25.8)*°	60.7 (34.2)*	*p* < 0.001
Hip extension	157.3 (42.3)	67.0 (38.4)*°	30.8 (36.5)*	*p* < 0.001
Hip flexion	196.9 (73.7)	100.4 (57.0)*	122.8 (48.5)*	*p* < 0.001

Abbreviations in alphabetic order: cm, centimeter; CP, cerebral palsy; DMD, Duchenne muscular dystrophy; GMFCS, gross motor function classification scale; IQR, interquartile range; kg, kilogram; m, meter; N, Newton; Nm, Newton meter; Nm/kg, Newton meter per kilogram body weight; TD, typically developing. Symbols represent significance according to the Mann Whitney U test with p < 0.0036: *TD-CP, *TD-DMD, °CP-DMD.

**FIGURE 3:**
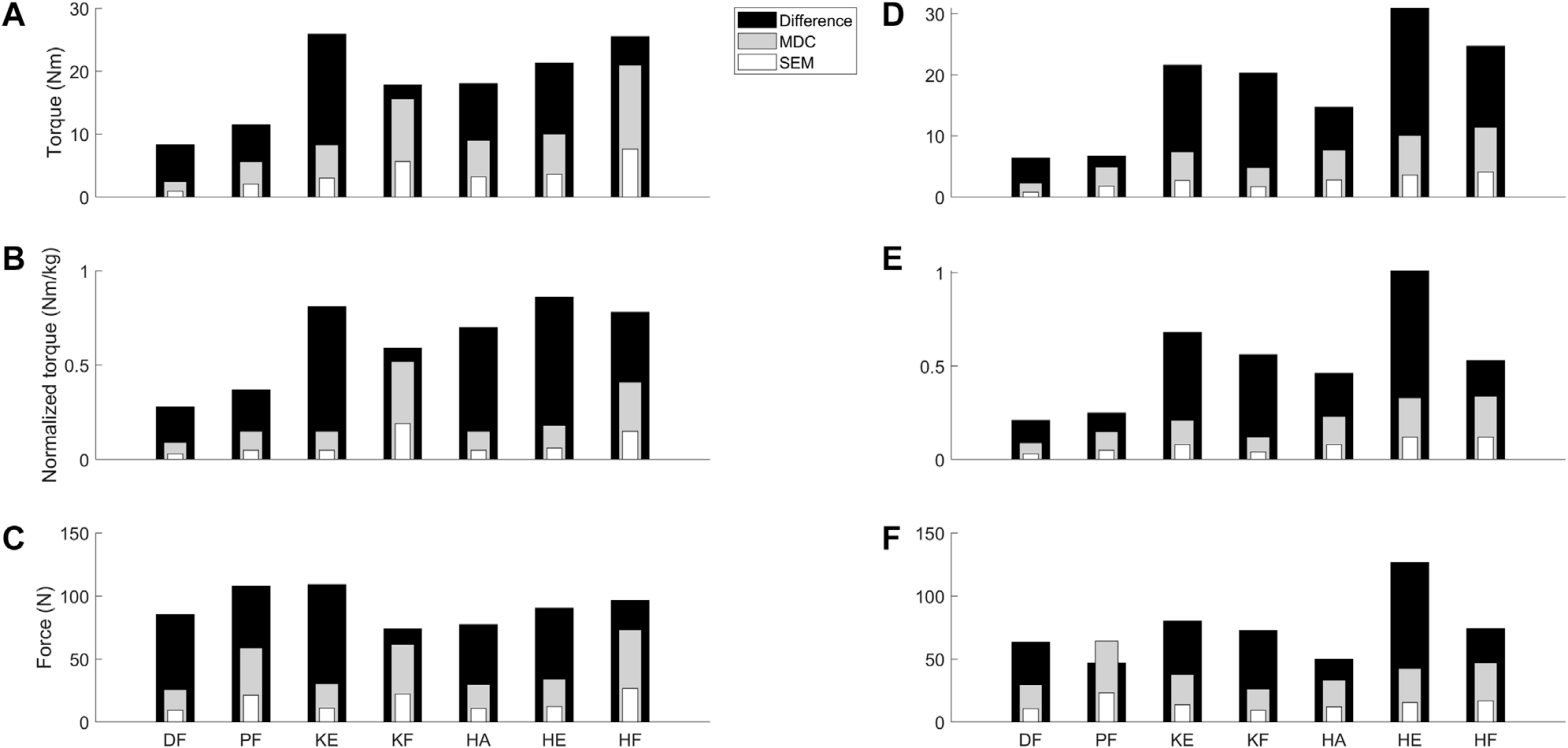
Visual representation to compare the absolute SEM (white) and MDC (grey) values as retrieved from the reliability study for the children with CP and DMD with the absolute differences between the median values of TD children and the clinical cohorts (black) from the validity study. Panel A–C visualize the data for torque **(A)**, normalized torque **(B)** and the force **(C)** in the children with CP. Panel D–F visualize the data for the torque **(D)**, normalized torque **(E)** and the force **(F)** in the children with DMD. Abbreviations in alphabetical order: CP, cerebral palsy; DF, dorsiflexion; DMD, Duchenne muscular dystrophy; HA, hip abduction; HE, hip extension; HF, hip flexion; KE, knee extension; KF, knee flexion; MDC, minimal detectable change; N, Newton; Nm, Newton meter; Nm/kg, Newton meter per kilogram body weight; PF, plantar flexion; SEM, standard error of measurement; TD, typically developing.

The absolute differences between the medians of the TD and CP cohort ranged between 8.3 Nm (DF) and 25.9 Nm (KE) for torque, and between 0.28 Nm/kg (DF) and 0.86 Nm/kg (HE) for normalized torque (Supplementary Table S2). The absolute differences for the CP cohort for torque and normalized torque were all above the absolute SEM and MDC of the CP cohort (Figures 3A–C). The absolute differences between the medians of the TD and DMD cohort ranged between 6.4 Nm (DF) and 30.9 Nm (HE) for torque, and between 0.21 Nm/kg (DF) and 1.01 Nm/kg (HE) for normalized torque (Supplementary Table S2). These absolute differences were all above the absolute SEM and MDC of the DMD cohort, except for PF force (Figures 3D–F). To limit the discussed parameters and outcomes, the comparison of the relative differences with SEM% and MDC% were included in Supplementary Table S2 and Supplementary Figure S1.

### 3.3 Part three: responsiveness

As descriptive results, the subject characteristics and the median values of the MVICs for the responsiveness study are presented in Table 5 for the CP and in Table 6 for the DMD cohort. In the CP cohort, the muscle strength increased after the strength intervention in all muscle groups (*p* < 0.003). The DMD cohort showed no statistically significant changes in muscle strength over the observed intervals. However, several trends in decreasing muscle strength appeared (*p* < 0.05).

**TABLE 5:** Subject characteristics, median MVICs of the first and second assessment and statistical results after the strength intervention of children with CP included in the responsiveness study.

	CP		
Subject information	Frequency or median (IQR)		
Number of participants	26		
Age (years)	8.1 (4.5)		
Weight (kg)	28.4 (15.2)		
Height (cm)	127.8 (24.9)		
GMFCS-level	I:17 II:6 III:3		

			Wilcoxon Rank test
	A1	A2	*p*-value
Primary parameter: Torque (Nm)			
Dorsiflexion	2.0 (2.1)	3.2 (2.6)	*p* = 0.003
Plantar flexion	5.1 (5.8)	10.3 (7.3)	*p* < 0.001
Knee extension	12.4 (20.1)	20.0 (17.4)	*p* < 0.001
Knee flexion	8.4 (11.1)	18.4 (15.6)	*p* < 0.001

Primary parameter: Normalized torque (Nm/kg)			
Dorsiflexion	0.08 (0.7)	0.11 (0.12)	*p* = 0.003
Plantar flexion	0.19 (0.16)	0.33 (0.17)	*p* < 0.001
Knee extension	0.44 (0.57)	0.62 (0.42)	*p* = 0.003
Knee flexion	0.32 (0.32)	0.58 (0.39)	*p* < 0.001

Secondary parameter: Force (N)			
Dorsiflexion	24.7 (19.3)	38.0 (29.5)	*p* = 0.001
Plantar flexion	59.4 (56.0)	125.3 (71.6)	*p* < 0.001
Knee extension	62.0 (86.2)	95.7 (68.5)	*p* = 0.003
Knee flexion	40.1 (44.1)	81.7 (61.9)	*p* < 0.001

Abbreviations in alphabetical order: A1, assessment one; A2, assessment two; CP, cerebral palsy; GMFCS, gross motor function classification scale; IQR, interquartile range; kg, kilogram; cm, centimeter; N, Newton; Nm, Newton meter; Nm/kg, Newton meter per kilogram body weight.

**TABLE 6:** Subject characteristics, median MVICs of the first and second assessment and statistical results for the 1-year and 2-year follow-up period of boys with DMD included in the responsiveness study.

	DMD 1 year interval			DMD 2 year interval		
	Frequency or median (IQR)			Frequency or median (IQR)		
Number of participants	8 (i.e., 13 pairs of measurements)			6 (i.e., 6 pairs of measurements)		
Age (years)	11.0 (2.8)			9.8 (3.1)		
Weight (kg)	36.7 (10.7)			33.1 (11.0)		
Height (cm)	127.5 (11.0)			126.7 (14.0)		

	A1	A2	Wilcoxon Rank test (*p*-value)	A1	A2	Wilcoxon Rank test (*p*-value)
Primary parameter: Torque (Nm)						
Dorsiflexion	4.7 (1.2)	4.0 (1.7)	*p* = 0.087	4.8 (0.7)	3.7 (1.6)	*p* = 0.075
Plantar flexion	7.2 (5.5)	9.4 (7.5)	*p* = 0.701	7.1 (5.0)	5.9 (4.4)	*p* = 0.600
Knee extension	12.2 (8.9)	10.1 (7.1)	*p* = 0.133	13.2 (11.4)	8.9 (7.4)	*p* = 0.075
Knee flexion	9.6 (6.3)	9.4 (6.6)	*p* = 0.507	10.3 (5.8)	7.6 (5.85)	*p* = 0.463
Hip abduction	12.1 (9.0)	8.9 (5.6)	*p* = 0.087	12.4 (9.7)	9.9 (4.6)	*p* = 0.028
Hip extension	4.3 (6.1)	3.7 (3.6)	*p* = 0.345	4.9 (12.3)	2.7 (7.8)	*p* = 0.600
Hip flexion	24.3 (17.2)	20.5 (18.9)	*p* = 0.311	24.8 (24.5)	19.1 (27.4)	*p* = 0.249

Primary parameter: Normalized torque (Nm/kg)						
Dorsiflexion	0.14 (0.06)	0.10 (0.06)	*p* = 0.029	0.16 (0.04)	0.10 (0.04)	*p* = 0.028
Plantar flexion	0.25 (0.17)	0.23 (0.21)	*p* = 0.906	0.24 (0.16)	0.20 (0.14)	*p* = 0.207
Knee extension	0.34 (0.21)	0.26 (0.19)	*p* = 0.092	0.36 (0.34)	0.23 (0.26)	*p* = 0.043
Knee flexion	0.28 (0.18)	0.24 (0.14)	*p* = 0.220	0.32 (0.10)	0.22 (0.15)	*p* = 0.080
Hip abduction	0.33 (0.24)	0.25 (0.17)	*p* = 0.041	0.40 (0.32)	0.27 (0.15)	*p* = 0.028
Hip extension	0.10 (0.14)	0.10 (0.07)	*p* = 0.184	0.14 (0.35)	0.08 (0.16)	*p* = 0.249
Hip flexion	0.62 (0.50)	0.49 (0.45)	*p* = 0.235	0.75 (0.71)	0.58 (0.76)	*p* = 0.043

Secondary parameter: Force (N)						
Dorsiflexion	55.9 (16.1)	43.3 (15.2)	*p* = 0.046	53.4 (18.2)	39.3 (22.7)	*p* = 0.046
Plantar flexion	93.6 (79.9)	99.4 (85.4)	*p* = 0.917	85.2 (64.9)	71.3 (66.1)	*p* = 0.463
Knee extension	62.1 (47.5)	48.4 (34.7)	*p* = 0.075	65.7 (55.3)	43.4 (81.18)	*p* = 0.046
Knee flexion	48.1 (29.9)	48.0 (28.6)	*p* = 0.249	51.4 (25.9)	37.4 (23.6)	*p* = 0.345
Hip abduction	53.0 (33.4)	37.0 (25.2)	*p* = 0.046	53.4 (34.6)	38.2 (18.4)	*p* = 0.028
Hip extension	16.5 (24.2)	16.0 (13.9)	*p* = 0.279	23.1 (45.5)	10.7 (28.9)	*p* = 0.249
Hip flexion	98.6 (70.5)	90.1 (67.7)	*p* = 0.211	106.3 (94.5)	82.2 (99.2)	*p* = 0.173

Abbreviations in alphabetical order: A1, assessment one; A2, assessment two; DMD, Duchenne muscular dystrophy; IQR, interquartile range; kg, kilogram; cm, centimeter; N, Newton; Nm, Newton meter; Nm/kg, Newton meter per kilogram body weight.

**FIGURE 4:**
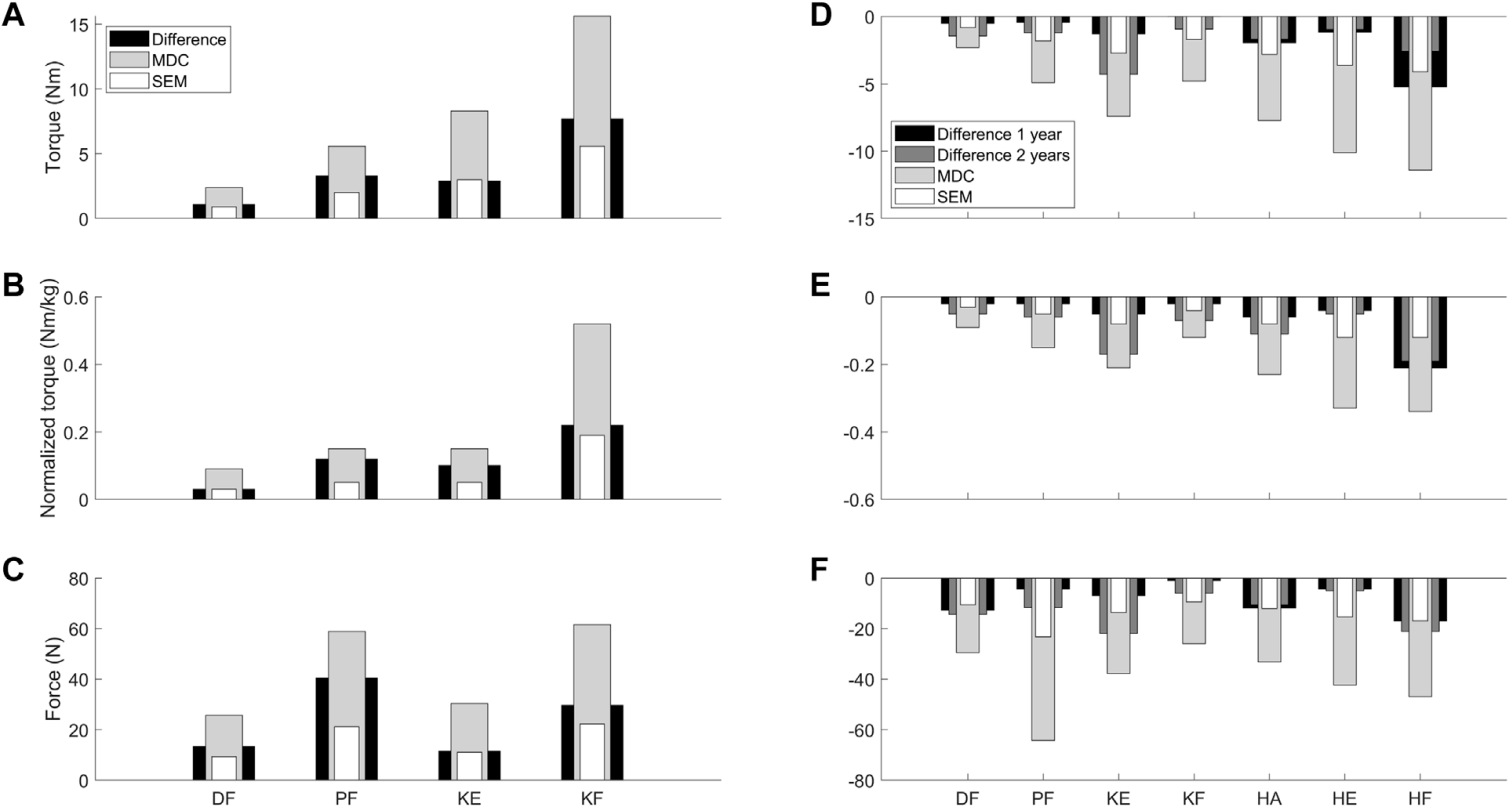
Visual representation to compare the absolute SEM (white) and MDC (light grey) values as retrieved from the reliability study for the children with CP and DMD with the median of the absolute differences between the two assessments (black = CP and 1 year interval DMD; dark grey = 2 year interval DMD) from the responsiveness study. Panel **A-C** visualize the data for torque **(A)**, normalized torque **(B)** and the force **(C)** in the children with CP. Panel **D-F** visualize the data for torque **(D)**, normalized torque **(E)** and the force **(F)** in the children with DMD. Abbreviations in alphabetical order: CP, cerebral palsy; DF, dorsiflexion; DMD, Duchenne muscular dystrophy; HA, hip abduction; HE, hip extension; HF, hip flexion; KE, knee extension; KF, knee flexion; MDC, minimal detectable change; N, Newton; Nm, Newton meter; Nm/kg, Newton meter per kilogram body weight; PF, plantar flexion; SEM, standard error of measurement.

The medians of the absolute differences observed in the CP cohort ranged between 1.1 Nm (DF) and 7.7 Nm (KF) for torque, and between 0.03 Nm/kg (DF) and 0.22 Nm/kg (KF) for normalized torque between the strength scores before and after training (Supplementary Table S3). All medians of the absolute differences were larger than the absolute SEM, except for KE torque and DF normalized torque, where the medians of the absolute increases were similar to the absolute SEM (Figures 4A–C). However, all medians of the absolute increases were lower than the absolute MDC values. In the DMD cohort, the absolute decreasing trend in HA torque was 1.66 Nm over an interval of 2 years (Supplementary Table S3). For normalized torque, DF, KE, HA, and HF showed trends in medians of the absolute decreases, ranging from 0.02 Nm/kg (DF) to 0.06 Nm/kg (HA) over an interval of 1 year and from 0.05 Nm/kg (DF) to 0.19 Nm/kg (HF) over an interval of 2 years. The detected trends for children with DMD revealed that the medians of the absolute decreases over an interval of 2 years exceeded the absolute SEM of the DMD cohort for normalized torque of DF, KE, HA, and HF (Figures 4D–F). For the other trends in DMD, i.e., DF and HA normalized torque over 1 year, and HA torque over 2 years, the median of the absolute decreases were smaller than the absolute SEM of the DMD cohort. All medians of the absolute decreases observed in boys with DMD were lower than the absolute MDC values. To limit the discussed parameters and outcomes, the relative differences with SEM% and MDC% from the responsiveness study were only included in Supplementary Table S3 and Supplementary Figure S2.

## 4 Discussion

The overall aim of the current study was to comprehensively determine the clinimetric properties of the instrumented strength assessment in a TD, CP, and DMD cohort for muscle groups around the ankle, knee and hip. To achieve this overall aim, three sub-aims were defined, and the study was divided in three parts, covering the reliability, the construct validity, and the responsiveness of the instrumented strength assessment.

### 4.1 Part one: reliability

Our hypothesis concerning the first sub-aim was only partly confirmed. Indeed, some strength measurements showed a lower reliability than expected. In general, the instrumented strength assessment showed moderate to excellent reliability in the CP and DMD cohort and poor to excellent reliability in the TD cohort. This difference between the clinical cohorts and the TD cohort may be partly explained by the difference in the assessors’ experience, since the human movement scientist and pediatric physiotherapist collected the assessments in the CP and DMD cohort, while the trained master students collected the assessments in the TD cohort. However, it can also be explained by the higher force producing capacity of TD participants, introducing a larger window for variation and resulting in more difficulties to limit compensations. A slightly lower than expected intra-rater reliability was observed for the torque of PF and HF in TD, of PF and KF in CP and of DF, PF and HA in DMD, which were all classified as moderate. For normalized torque, the reliability of DF and PF in TD, KF in CP and HA in DMD were classified as moderate, while the reliability of KE and measurements of the muscle groups around the hip were classified as poor in the TD cohort. There were no obvious differences between the reliability indices of TD children and the indices of the clinical cohorts. Yet, in both clinical cohorts, a wide range of CIs was observed, which can be attributed to the limited sample size. Hence, caution is needed in the interpretation of the results. In general, proximal muscle groups demonstrated a slightly better reliability than distal muscle groups in both patient groups. These results were in line with the findings in the study of Florence et al. (1992), who investigated the intra-rater reliability of manual muscle testing when assessing muscle strength in children with DMD. The study of Florence et al. (1992) showed a reliability range with ICCs from 0.65 to 0.93, with the proximal muscles presenting higher reliability values (Florence et al., 1992). In the study of Berry et al. (2004), results similar to the current study where found, while their set-up did not include fixation of the HHD. Berry et al. (2004) included 15 children diagnosed with CP to evaluate the intra- and intersession reliability of the HHD, based on bilateral measurements of KE, KF and HA, with a time interval of 4–14 days. Intra-rater ICCs of 0.840 or higher were found, except for the left KF (Berry et al., 2004). The current study confirmed these results for the KE and HA muscles, but not for the KF muscles, which showed a lower ICC value.

The current study results revealed that the inter-rater intrasession ICCs were higher than the intra-rater intersession ICCs in the TD cohort, pointing towards the potential impact of repositioning the child in the set-up and the potential different performance on different test days, which appears to be larger than the impact of different raters. However, the impact of training experience on the applied instrumented strength assessments should be further explored in future studies. Goudriaan et al. (2018a) investigated the intra-rater and inter-rater reliability of the instrumented strength assessment (within one session) and showed higher inter-rater ICCs compared to the intra-rater ICCs, whilst torque showed better ICCs than normalized torque (Goudriaan et al., 2018a). The current study confirmed these results, showing similar ranges of the inter- and intra-rater ICCs in the TD cohort, and added results for the hip joint and two clinical cohorts (Goudriaan et al., 2018a).

In the current study, normalization of TD torque data to body weight resulted in lower ICC-values with wider CIs. This is not surprising, since the variability of normalized torque data was smaller due to the known relationship between strength and body weight, resulting in lower ICC values. Indeed, it is known that the ICC depends on the variability of the data (Florence et al., 1992; Berry et al., 2004). The lower ICC values for PF torque (intra-rater: 0.626, inter-rater—intrasession: 0.234 and inter-rater—intersession: 0.397) could be explained by the complex nature of the PF movement. It is likely that children were still able to compensate in the knee and hip joint, knowing that fixating the ankle joint is complex. The results of the secondary parameters were in line with the findings of the primary parameters.

### 4.2 Part two: validity

Our hypothesis concerning the second sub-aim was confirmed. Significant differences were found for alle muscle groups, indicating that children with CP and DMD are overall weaker than TD children. The torque deficits in the CP cohort ranged from 49.6% for HF to 82.2% for DF (Supplementary Table S2), which is in agreement with previously reported ranges of lower limb muscle weakness (Dallmeijer et al., 2017; Hanssen et al., 2021). In the DMD cohort, the torque deficits ranged from 41.4% for PF to 80.3% for HE (Supplementary Table S2). Similar DF and KF strength deficits were found in previous literature, while the PF strength deficit was smaller in the current study (Mathur et al., 2010; Lerario et al., 2012; Wokke et al., 2014; Goudriaan et al., 2018b; Vandekerckhove et al., 2020). The KE torque deficit from the current study was in agreement with previously reported results from our research group (Goudriaan et al., 2018b; Vandekerckhove et al., 2020). While larger deficits for the KE strength have been found by other research groups using different strength assessments (Mathur et al., 2010; Lerario et al., 2012; Wokke et al., 2014). Following the approach of Buckon et al. (2016), who took the control data of Hébert et al. (2015) into account, the hip torque deficits from the current study were also in agreement with previous findings (Hébert et al., 2015; Buckon et al., 2016). However, statistically significant differences do not automatically prove relevant differences. The latter was evaluated by comparing the absolute differences between the TD and the two clinical cohorts with the absolute SEM and MDC values. The observed absolute differences between the median of the pathological cohorts and TD cohort exceeded the absolute MDC values in every subcategory, except for PF force in the DMD cohort. Overall, these findings prove the construct validity of the instrumented strength assessment. Future research is necessary to expand the findings of the validity part of this study to other clinical populations to strengthen its generalizability. Future studies should also compare the observed between-groups differences to minimally clinical important differences, for example by taking differences in muscle strength between pathological subgroups based on GMFCS-level, based on different gait patterns, or based on participation or quality of life scores as a reference. 

### 4.3 Part three: responsiveness

Our hypothesis concerning the third sub-aim was only partly confirmed, since all absolute differences were smaller than the MDC values in CP and DMD. After strength training, the strength in all muscle groups increased significantly in the children with CP. While most of the absolute increases exceeded the absolute SEM values, they were smaller than the absolute MDC values. Unfortunately, the hip joint was not included in the strength training protocol of the CP children. With this additional proof of the effectiveness of strength training in children with CP and the availability of the clinimetric properties of strength assessments, future studies could expand the protocol to the hip muscle groups, especially since these muscles showed significant weakness (Table 4). For the DMD children, no significant differences in muscle strength could be observed within a time interval of 1 or 2 years. Yet, DF, and HA MVICs tended to decrease over the observed interval (1 and 2 years). In addition, a tendency towards a decrease in KE and HF MVICs was detected, only over an interval of 2 years. The absolute decreases in the DMD cohort were all smaller than the absolute MDC values, however, the absolute decreases in DF, KE, HA, and HF normalized torque over 2 years were larger than the absolute SEM values. This suggests a shortness in sensitivity of the instrumented strength assessment for the DMD cohort when evaluating muscle strength with a time interval of 1 or 2 years. Yet, it should be noted that the sample of boys with DMD was still limited, which may have caused a lack of power. The included sample size in the DMD cohort was limited due to the rarity of the disease combined with the strict inclusion criteria in part three. Moreover, the sample showed a large heterogeneity, e.g., differences in underlying gene mutation (four boys: deletion; two boys: point mutation; one boy: in frameshift mutation and one boy: nonsense mutation), participation in clinical trials (50% participated in clinical trials; one boy: ataluren and three boys: givinostat) and corticosteroids dosage (100% calcort; dosage ranged between 15 and 21 mg), which might partly explain the lack of significant change in the MVICs. Nevertheless, clinical tendencies towards increasing muscle weakness could be observed within the DMD results, ranging from 12.5% (HA torque) to 39% (KE normalized torque) over an interval of 2 years (Supplementary Table S3). McDonald et al. (1995) reported a decrease in KE isometric strength from 50% of control data at 6 years old to 0% at 12 years old (McDonald et al., 1995). This corresponds to a relative decrease in KE muscle strength of 33.3% in 2 years and therefore, is in agreement with the current study. However, careful study of the individual results showed that for the torque and normalized torque parameters, 32% of the measurement pairs over 1 year and 17% of the measurement pairs over 2 years presented increases in muscle strength between the two assessments, suggesting that these children with DMD did not yet lose muscle strength. This is surprising, since the boys with DMD were selected based on an age criterium of 7.5 years and a clinically meaningful decrease in the 6 MWT. The interaction between functional deterioration and strength loss in the natural history of the disease should be further investigated.

### 4.4 Limitations

In general, strength assessments in pediatric cohorts, especially in children with neurological and neuromuscular diseases, are challenging. While the goal was to obtain three well executed actual trials, not all children with CP or DMD were able to fulfil this task due to fatigue. It must also be noted that the practical applicability of the instrumented strength assessment can be challenging in clinical practice in comparison to the HHD, considering the size of the external structure and the alterations that must be made during the measurements to adapt the instrumented strength assessment to the different muscle groups. On the other hand, all the joint movements can be assessed with the patient in the same position, avoiding excessive repositioning of the patient. In addition, the results revealed that the instrumented strength assessment can be considered reliable in TD children and children with CP and DMD, albeit these limitations. Despite the applied fixations, compensation could not be entirely avoided when performing the MVICs. For example, during PF, co-contraction in knee and hip joint could still be observed, even after verbal instructions, and when performing HF, the waistbelt was not always sufficiently tight to prevent children from moving in the chair. A possible solution might be to replace the waistbelt with a type of safety harness, fixating both the trunk and pelvis and thereby further limiting compensations when producing forces around the hip. Electromyography could be applied to assess muscle activity during MVICs and quantify potential compensations, co-contractions or synergies during MVICs. Another important consideration of the applied study protocol was the measurement of the segment lengths from which the lever arms were calculated. The manual measurement of the segment lengths using a tape measure may be sensitive to errors. It is important to take into account that small errors in these measurements could have influenced the lever arm which was necessary to calculate torque and normalized torque. Furthermore, the applied joint positions did not correspond with common test protocols. Future test positions can be chosen based on the populations, the comparison with other daily life activities and the available reference data.

## 5 Conclusion

A concluding overview of all investigated clinimetric properties of the instrumented strength assessment and its usability is given in Table 7. The instrumented strength assessment showed moderate to excellent reliability results and proved sufficiently reliable to confirm the known-group validity for both clinical cohorts. The reliability results in the TD cohort indicated the need for further standardization of the strength assessments at the hip and all cohorts indicated this need for assessment of PF strength. Where the instrumented strength assessment was able to detect the responsiveness of children with CP after a strength intervention, more research is necessary to determine the responsiveness of DMD regarding their natural decline. Consequently, as highlighted in the overview in Table 7, the assessment is ready to be used in clinical studies on children with CP, although the responsiveness around the hip joint remains to be determined, whereas further research on the responsiveness of the instrumented strength assessment for boys with DMD is needed. Thereby, the use of a larger sample size, a more homogenous group of children with DMD and a further improvement of the standardization could improve the results. In addition, future research could define the minimal important clinical difference of the assessment as well as transfer the assessment to other pediatric patient populations.

**TABLE 7:** Overview and overall conclusion of the clinimetric properties of the instrumented strength assessment in Typically developing children and children with cerebral palsy and Duchenne muscular dystrophy.

TYPICALLY DEVELOPING CHILDREN					
	Intra-rater reliability	Inter-rater inter-session	Inter-rater intra-session	Overall conclusion	Advice
Dorsiflexion	Excellent	Excellent	Excellent	USE +++	Ready to be used.
Plantar flexion	Moderate	Poor	Poor	Limited USE- -	More research needed to optimize assessment standardization
Knee extension	Good	Excellent	Good	USE +++	Ready to be used.
Knee flexion	Excellent	Good	Good	USE +++	Ready to be used.
Hip abduction	Good	Good	Good	USE +++	Ready to be used.
Hip extension	Good	Moderate	Good	USE ++	Ready to be used, with special attention on assessment standardization
Hip flexion	Moderate	Moderate	Good	Partial USE +	Careful use, with special attention on assessment standardization

CHILDREN WITH CEREBRAL PALSY					
	Intra-rater reliability	Validity	Response	Overall conclusion	Advice
Dorsiflexion	Good	Excellent	Good	USE +++	Ready to be used
Plantar flexion	Moderate	Excellent	Good	USE ++	Ready to be used, but with special attention on assessment standardization
Knee extension	Excellent	Excellent	Good	USE +++	Ready to be used
Knee flexion	Moderate	Excellent	Good	USE ++	Ready to be used, but with special attention on assessment standardization
Hip abduction	Good	Excellent	Unknown	USE ++	Ready to be used for group comparison. Unknown for response.
Hip extension	Excellent	Excellent	Unknown	USE ++	Ready to be used for group comparison. Unknown for response.
Hip flexion	Good	Excellent	Unknown	USE ++	Ready to be used for group comparison. Unknown for response.

CHILDREN WITH DUCHENNE MUSCULAR DYSTROPHY					
	Intra-rater reliability	Validity	Response	Overall conclusion	Advice
Dorsiflexion	Moderate	Excellent	Moderate	Partial USE +	Careful use. More research required to strengthen the evaluation of response.
Plantar flexion	Moderate	Good	Absent	Limited USE +/-	Careful use, with special attention on standardization. Not ready to evaluate response.
Knee extension	Excellent	Excellent	Moderate	USE ++	Ready to be used, but more research is required to strengthen the evaluation of response.
Knee flexion	Good	Excellent	Absent	Partial USE +	Ready to be used for group comparison. Not ready to evaluate response.
Hip abduction	Moderate	Excellent	Moderate	Partial USE +	Careful use. More research required to strengthen the evaluation of response.
Hip extension	Good	Excellent	Absent	Partial USE +	Ready to be used for group comparison. Not ready to evaluate response.
Hip flexion	Good	Excellent	Moderate	USE ++	Ready to be used, but more research required to strengthen the evaluation of response.

The following scoring was applied. Reliability: the reliability of the torque parameters are reported following Koo and Li. (2016): poor: ICC≤ 0.500, moderate: ICC = 0.501–0.750, good: ICC = 0.751–0.900 and excellent: ICC>0.900. Validity or Responsiveness: absent = no significant p-values [p >0.0036 and p>0.0063 (CP, responsiveness)] and no trends (p>0.05); poor = trend (p<0.05) but absolute differences (i.e. absolute difference between median of clinical cohort and TD, cohort for validity and median of all absolute differences between assessment one and two per participant for responsiveness) smaller than SEM, and MDC; moderate = trend (p<0.05) and absolute differences larger than SEM, but smaller than MDC; good = trend (p<0.05) and absolute differences larger than SEM, and MDC, or significant p-value (p<0.0036 and p < 0.0063 (CP, responsiveness)) and absolute differences larger than SEM, but not larger than MDC; excellent = significant p-value [p < 0.0036 and p < 0.0063 (CP, responsiveness)] and absolute differences larger than SEM, and MDC. The overall conclusion was based on a summation of the first three columns of the table (for TD: all reliability assessments and for the clinical cohorts: reliability, validity and responsiveness). First, good and excellent was scored as +, moderate as and poor and absent as − per column and then, summed for the overall conclusion. If the overall conclusion is +++ or ++, the instrumented strength assessment is recommended to be used to assess the strength of the corresponding muscle group. If the overall conclusion is +, partial use is recommended. If the overall conclusion is +/−, −, −− or −−−, limited use is recommended. A more detailed advice is described in the last column. Abbreviations in alphabetical order: CP, cerebral palsy; DMD, Duchenne muscular dystrophy; MDC, minimal detectable change; SEM, standard error of measurement; TD, typically developing.

## Data Availability

The datasets presented in this study can be found in online repositories. The names of the repository/repositories and accession number(s) can be found in the article/Supplementary Material.

## Supplementary material

The datasets presented in this study can be found online at: https://dx.doi.org/10.6084/m9.figshare.18427295.
