# Erratum: Instrumented strength assessment in typically developing children and children with a neural or neuromuscular disorder: A reliability, validity and responsiveness study

**DOI:** 10.3389/fphys.2022.1120014

**Published:** 2022-12-23

**Authors:** Frontiers Production Office

**Affiliations:** Frontiers Media SA, Lausanne, Vaud, Switzerland

**Keywords:** cerebral palsy, duchenne musclar dystrophy, muscle weakness, instrumented strength assessment, clinimetric properties, reliability, validity, responsiveness

Due to a production error, there were several errors in the tables in the article.

In Table 1, some of the abbreviations in the footnote were missing. The abbreviations read as “Abbreviations in alphabetical order: BTX, botulinum toxin infiltration; CP, cerebral palsy; DMD, Duchenne muscular dystrophy; GMFCS, gross motor function classification system; TD, typical developing”. The correct abbreviations list is “BTX, botulinum toxin infiltration; CP, cerebral palsy; DMD, Duchenne muscular dystrophy; GMFCS, gross motor function classification system; m, meter; TD, typically developing”.

In Tables 2, 3, 4, 5, and 6, the word “Plantarflexion” is incorrect. It should read as “Plantar flexion”.

In Table 2, the bottom part of the header read as “Frequency or median (IQR)”. The correct header is “Median (IQR) ICC (95%IC) SEM MDC)”.

In Table 3, the title was incorrectly written as “Intra-rater intersession reliability results of the TD children as well as children with CP and DMD included in the reliability study.” The correct title is “Inter-rater intrasession and intersession reliability results for the TD children included in the reliability study”.

In Table 3, the numbers under the headings “TD: Inter-rater intrasession reliability (A1-A2)” and “TD: Inter-rater intrasession reliability (A1-A3)” only appeared once. These numbers should have appeared twice.

In Table 3, some of the abbreviations in the footnote were missing. The abbreviations read as “Abbreviations in alphabetical order: CI, confidence interval; cm, centimeter; ICC, intraclass correlation coefficient; IQR, interquartile range; kg, kilogram; MDC, minimal detectable change; N, newton; Nm, newton meters; Nm/kg, newton meters per kilogram; SEM, standard error of measurement; TD, typically developing; Green, excellent to good reliability; Blue, moderate reliability; Red, poor reliability”. The correct abbreviation list is “Abbreviations in alphabetical order: A1, assessment one; A2, assessment two; A3, assessment three; CI, confidence interval; cm, centimeter; ICC, intraclass correlation coefficient; IQR, interquartile range; kg, kilogram; MDC, minimal detectable change; N, newton; Nm, newton meters; Nm/kg, newton meters per kilogram; SEM, standard error of measurement; TD, typically developing; Green, excellent to good reliability; Blue, moderate reliability; Red, poor reliability”.

In Table 4, the second sub-heading read as “Normalized torque (Nm/kg)”. The correct heading is “Primary parameter: Normalized torque (Nm/kg)”

In Table 4, the third sub-heading read as “Secondary parameters Force (N)”. The correct heading is “Secondary parameter: Force (N)”

In Table 4, some of the abbreviations in the footnote were missing. The abbreviations read as “Abbreviations in alphabetic order: CP, cerebral palsy; DMD, Duchenne muscular dystrophy; GMFCS, gross motor function classification scale; IQR, interquartile range; kg, kilogram; m, < meter; N, newton; Nm, Nm; Nm/kg; Nm, per kilogram body weight; TD, typical developing. Symbols represent significance according to the Mann Whitney U test with p 0.0036: *TD-CP, *TD-DMD, CP-DMD.” The correct abbreviation list is “cm, centimeter; CP, cerebral palsy; DMD, Duchenne muscular dystrophy; GMFCS, gross motor function classification scale; IQR, interquartile range; kg, kilogram; m, meter; N, Newton; Nm, Newton meter; Nm/kg, Newton meter per kilogram body weight; TD, typically developing. Symbols represent significance according to the Mann Whitney U test with *p* < 0.0036: *TD-CP, *TD-DMD, CP-DMD”.

In Table 5, the second sub-heading read as “Primary parameters torque (Nm)”. The correct heading is “Primary parameter: Torque (Nm)”.

In Table 5, the third sub-heading read as “Normalized torque (Nm/kg)”. The correct heading is “Primary parameter: Normalized torque (Nm/kg)”.

In Table 5, the fourth sub-heading read as “Secondary parameters Force (N)”. The correct heading is “Secondary parameter: Force (N)”.

In Table 5, some of the abbreviations in the footnote were missing. The abbreviations read as “Abbreviations in alphabetical order: CP, cerebral palsy; GMFCS, gross motor function classification scale; IQR, interquartile range; kg, kilogram; cm, centimeter; N, newton; Nm, Nm; Nm/kg, Nm per kilogram body weight”. The correct abbreviations list is “Abbreviations in alphabetical order: A1, assessment one; A2, assessment two; CP, cerebral palsy; GMFCS, gross motor function classification scale; IQR, interquartile range; kg, kilogram; cm, centimeter; N, Newton; Nm, Newton meter; Nm/kg, Newton meter per kilogram body weight”.

In Table 6, some of the abbreviations in the footnote were missing. The abbreviations read as “Abbreviations in alphabetical order: DMD, Duchenne muscular dystrophy; IQR, interquartile range; kg, kilogram; cm, centimeter; N, newton; Nm, Nm; Nm/kg, Nm per kilogram body weight”. The correct abbreviations list is “Abbreviations in alphabetical order: A1, assessment one; A2, assessment two; DMD, Duchenne muscular dystrophy; IQR, interquartile range; kg, kilogram; cm, centimeter; N, Newton; Nm, Newton meter; Nm/kg, Newton meter per kilogram body weight”.

In Table 7, the footnotes were not implemented correctly. The footnote read as “The following scoring was applied. Reliability: the reliability of the torque parameters are reported following Koo and Li. (2016): poor = ICC<0.500 =, moderate = ICC, of 0.500–0.750 =, good = ICC, of 0.750–0.900 and excellent = ICC>0.900. Validity or Responsiveness: absent = no significant *p*-values [*p* > 0.0036 and *p* > 0.0063 (CP, responsiveness)] and no trends (*p* > 0.05); poor = trend (*p* < 0.05) but absolute differences (i.e., absolute difference between median of clinical cohort and TD, cohort for validity and median of all absolute differences between assessment one and two per participant for responsiveness) smaller than SEM, and MDC; moderate = trend (*p* < 0.05) and absolute differences larger than SEM, but smaller than MDC; good = trend (*p* < .05) and absolute differences larger than SEM, and MDC, or significant *p*-value [*p* < 0.0036 and *p* < 0.0063 (CP, responsiveness)] and absolute differences larger than SEM, but not larger than MDC; excellent = significant *p*-value [*p* < 0.0036 and *p* < 0.0063(CP, responsiveness)] and absolute differences larger than SEM, and MDC. The overall conclusion was based on a summation of the first three columns of the table (for TD: all reliability assessments and for the clinical cohorts: reliability, validity and responsiveness). First, good and excellent was scored as +, moderate as and poor and absent as–per column and then, summed for the overall conclusion. If the overall conclusion is +++ or ++, the instrumented strength assessment is recommended to be used to assess the strength of the corresponding muscle group. If the overall conclusion is +, partial use is recommended. If the overall conclusion is +/-, -, – or —, limited use is recommended. A more detailed advice is described in the last column.” The correct footnote is “Reliability: the reliability of the torque parameters are reported following Koo and Li. (2016): poor: ICC≤ 0.500, moderate: ICC = 0.501–0.750, good: ICC = 0.751–0.900 and excellent: ICC > 0.900. Validity or Responsiveness: absent = no significant *p*-values [*p* > 0.0036 and *p* > 0.0063 (CP, responsiveness)] and no trends (*p* > 0.05); poor = trend (*p* < 0.05) but absolute differences (i.e., absolute difference between median of clinical cohort and TD, cohort for validity and median of all absolute differences between assessment one and two per participant for responsiveness) smaller than SEM, and MDC; moderate = trend (*p* < 0.05) and absolute differences larger than SEM, but smaller than MDC; good = trend (*p* < 0.05) and absolute differences larger than SEM, and MDC, or significant *p*-value (*p* < 0.0036 and *p* < 0.0063 (CP, responsiveness)) and absolute differences larger than SEM, but not larger than MDC; excellent = significant *p*-value [*p* < 0.0036 and *p* < 0.0063 (CP, responsiveness)] and absolute differences larger than SEM, and MDC. The overall conclusion was based on a summation of the first three columns of the table (for TD: all reliability assessments and for the clinical cohorts: reliability, validity and responsiveness). First, good and excellent was scored as +, moderate as and poor and absent as–per column and then, summed for the overall conclusion. If the overall conclusion is +++ or ++, the instrumented strength assessment is recommended to be used to assess the strength of the corresponding muscle group. If the overall conclusion is +, partial use is recommended. If the overall conclusion is +/-, -, -- or ---, limited use is recommended. A more detailed advice is described in the last column. Abbreviations in alphabetical order: CP, cerebral palsy; DMD, Duchenne muscular dystrophy; MDC, minimal detectable change; SEM, standard error of measurement; TD, typically developing.

The publisher apologizes for the mistake. The original article has been updated.

