# Supplementary material for: Instrumented strength assessment in typically developing children and children with a neural or neuromuscular disorder: A reliability, validity and responsiveness study
Source: Front Physiol. 2022 Oct 19;13:855222. doi: 10.3389/fphys.2022.855222 (PMC9627606; doi:10.3389/fphys.2022.855222)
Supplement: Supplementary file 1 [file DataSheet1.pdf]

Supplementary table 1: Relative SEM and MDC from the intra-rater intersession reliability of the three cohorts (i.e., TD, CP and DMD) and from the inter-rater intrasession and inter-rater intersession reliability of the TD cohort.

	**Intra-rater intersession**						**Inter-rater intrasession**		**Inter-rater intersession**	
	**TD**		**CP**		**DMD**		**TD**			
	**%SEM**	**%MDC**	**%SEM**	**%MDC**	**%SEM**	**%MDC**	**%SEM**	**%MDC**	**%SEM**	**%MDC**
**Primary parameters Torque (Nm)**										
Dorsiflexion	16.7	46.2	24.7	68.5	22.8	63.2	14.3	39.5	15.4	42.8
Plantarflexion	21.3	59.2	30.5	84.6	20.7	57.4	43.3	120.0	32.5	90.0
Knee extension	20.1	55.6	22.4	62.0	17.5	48.6	11.1	30.7	23.7	65.7
Knee flexion	12.4	34.4	35.1	97.3	11.9	32.9	17.5	48.5	21.8	60.3
Hip abduction	14.0	38.8	34.3	95.0	16.9	46.8	14.4	39.9	16.7	46.2
Hip extension	23.8	66.0	15.9	44.2	17.6	48.7	25.2	69.8	20.9	57.9
Hip flexion	29.3	81.2	24.3	67.4	13.5	37.3	22.3	61.9	19.1	53.1
**Normalized torque (Nm/kg)**										
Dorsiflexion	10.8	29.8	39.8	110.4	28.0	77.5	11.9	33.1	10.7	29.8
Plantarflexion	24.1	66.7	39.0	108.2	22.8	63.2	31.8	88.1	29.3	81.3
Knee extension	18.0	49.9	18.1	50.1	18.8	52.2	27.1	75.0	25.8	71.4
Knee flexion	9.5	26.4	52.9	146.5	11.7	32.4	17.8	49.3	18.6	51.6
Hip abduction	15.1	41.8	21.6	59.8	18.9	52.5	13.0	36.0	17.6	48.8
Hip extension	19.5	54.1	14.2	39.2	22.0	61.0	21.7	60.3	20.6	57.2
Hip flexion	23.0	63.7	17.2	47.7	15.1	42.0	19.9	55.2	18.4	51.0
**Secondary parameters Force (N)**										
Dorsiflexion	13.0	35.9	25.9	71.9	22.2	61.7	13.6	37.6	15.2	42.0
Plantarflexion	22.6	62.6	29.6	82.1	22.4	62.1	39.2	108.8	31.2	86.4
Knee extension	19.5	54.1	18.4	51.1	17.2	47.5	7.4	20.5	17.9	49.6
Knee flexion	10.4	28.9	31.8	88.3	13.6	37.6	19.2	53.1	20.4	56.5
Hip abduction	14.6	40.5	30.1	83.4	18.0	50.0	13.4	37.1	17.8	49.5
Hip extension	23.4	65.0	13.6	37.6	15.6	43.3	23.0	63.8	20.3	56.2
Hip flexion	23.8	65.8	20.6	57.1	12.6	34.8	19.2	53.3	16.1	44.6

Abbreviations in alphabetic order: CP = cerebral palsy, DMD = Duchenne muscular dystrophy, MDC = minimal detectable change, N = newton, Nm = newton meters, Nm/kg = newton meters per kilogram; SEM = standard error of measurement, TD = typically developing.

Supplementary table 2: Absolute and relative (i.e., relative calculated as absolute difference relative to median data of TD cohort) differences between median data of TD children and two clinical cohorts, i.e., children with CP and DMD, from the validity study, in comparison to the absolute and relative SEM and MDC of the clinical cohorts, from the reliability study.

	CP						DMD					
	**A. Diff. TD-CP**	**SEM**	**MDC**	**R. Diff. TD-CP**	**%SEM**	**%MDC**	**A. Diff. TD-DMD**	**SEM**	**MDC**	**R. Diff. TD-DMD**	**%SEM**	**%MDC**
**Primary parameters Torque (Nm)**												
Dorsiflexion	8.3	0.9	2.4	82.2	24.7	68.5	6.4	0.8	2.3	63.4	22.8	63.2
Plantarflexion	11.5	2.0	5.6	71.0	30.5	84.6	6.7	1.8	4.9	41.4	20.7	57.4
Knee extension	25.9	3.0	8.3	68.0	22.4	62.0	21.6	2.7	7.4	56.7	17.5	48.6
Knee flexion	17.8	5.6	15.6	60.1	35.1	97.3	20.3	1.7	4.8	68.6	11.9	32.9
Hip abduction	18.0	3.2	9.0	67.9	34.3	95.0	14.7	2.8	7.7	55.5	16.9	46.8
Hip extension	21.3	3.6	10.0	55.3	15.9	44.2	30.9	3.6	10.1	80.3	17.6	48.7
Hip flexion	25.5	7.6	21.0	49.6	24.3	67.4	24.7	4.1	11.4	48.1	13.5	37.3
**Normalized torque (Nm/kg)**												
Dorsiflexion	0.28	0.03	0.09	84.8	39.8	110.4	0.21	0.03	0.09	63.6	28.0	77.5
Plantarflexion	0.37	0.05	0.15	71.2	39.0	108.2	0.25	0.05	0.15	48.1	22.8	63.2
Knee extension	0.81	0.05	0.15	68.6	18.1	50.1	0.68	0.08	0.21	57.6	18.8	52.2
Knee flexion	0.59	0.19	0.52	66.3	52.9	146.5	0.56	0.04	0.12	62.9	11.7	32.4
Hip abduction	0.70	0.05	0.15	77.8	21.6	59.8	0.46	0.08	0.23	51.1	18.9	52.5
Hip extension	0.86	0.06	0.18	68.8	14.2	39.2	1.01	0.12	0.33	80.8	22.0	61.0
Hip flexion	0.78	0.15	0.41	54.5	17.2	47.7	0.53	0.12	0.34	37.1	15.1	42.0
**Secondary parameters Force (N)**												
Dorsiflexion	85.4	9.3	25.7	80.3	25.9	71.9	63.4	10.6	29.5	59.6	22.2	61.7
Plantarflexion	108.0	21.2	58.9	66.7	29.6	82.1	46.9	23.2	64.3	29.0	22.4	62.1
Knee extension	109.0	11.0	30.4	67.1	18.4	51.1	80.1	13.6	37.8	49.3	17.2	47.5
Knee flexion	73.9	22.2	61.6	60.7	31.8	88.3	72.8	9.4	26.0	59.8	13.6	37.6
Hip abduction	77.4	10.7	29.7	70.0	30.1	83.4	49.8	12.0	33.2	45.1	18.0	50.0
Hip extension	90.3	12.3	34.2	57.4	13.6	37.6	126.5	15.3	42.4	80.4	15.6	43.3
Hip flexion	96.5	26.5	73.3	49.0	20.6	57.1	74.1	16.9	46.9	37.6	12.6	34.8

Abbreviations in alphabetic order: A. Diff. = absolute difference; CP = cerebral palsy, DMD = Duchenne muscular dystrophy, MDC = minimal detectable change, N = newton, Nm = newton meters, Nm/kg = newton meters per kilogram; R. Diff. = relative difference, SEM = standard error of measurement, TD = typically developing.

Supplementary table 3: The medians of the absolute and relative (i.e., absolute difference relative to the first assessment) differences between the two assessments of the children with CP and DMD, from the responsiveness study, in comparison to the absolute and relative SEM and MDC of the clinical cohorts, from the reliability study.

	CP						DMD							
	**A. Diff.**	**SEM**	**MDC**	**R. Diff.**	**%SEM**	**%MDC**	**A. Diff. 1 year**	**A. Diff. 2 years**	**SEM**	**MDC**	**R. Diff. 1 year**	**R. Diff. 2 years**	**%SEM**	**%MDC**
**Primary parameters Torque (Nm)**														
Dorsiflexion	1.1	0.9	2.4	43.2	24.7	68.5	-0.5	-1.4	0.8	2.3	-11.8	-29.0	22.8	63.2
Plantarflexion	3.3	2.0	5.6	74.0	30.5	84.6	-0.4	-1.2	1.8	4.9	-6.2	-17.8	20.7	57.4
Knee extension	2.9	3.0	8.3	28.8	22.4	62.0	-1.3	-4.3	2.7	7.4	-12.1	-29.0	17.5	48.6
Knee flexion	7.7	5.6	15.6	72.1	35.1	97.3	0.0	-0.9	1.7	4.8	0.3	-8.7	11.9	32.9
Hip abduction							-2.0	-1.7	2.8	7.7	-16.7	-12.5	16.9	46.8
Hip extension							-1.2	-1.0	3.6	10.1	-29.0	-37.4	17.6	48.7
Hip flexion							-5.2	-2.6	4.1	11.4	-23.5	-16.7	13.5	37.3
**Normalized torque (Nm/kg)**														
Dorsiflexion	0.03	0.03	0.09	40.8	39.8	110.4	-0.02	-0.05	0.03	0.09	-16.1	-35.4	28.0	77.5
Plantarflexion	0.12	0.05	0.15	72.6	39.0	108.2	-0.02	-0.06	0.05	0.15	-11.2	-31.4	22.8	63.2
Knee extension	0.10	0.05	0.15	23.4	18.1	50.1	-0.05	-0.17	0.08	0.21	-20.7	-39.0	18.8	52.2
Knee flexion	0.22	0.19	0.52	70.6	52.9	146.5	-0.02	-0.07	0.04	0.12	-7.0	-25.3	11.7	32.4
Hip abduction							-0.06	-0.11	0.08	0.23	-24.2	-28.5	18.9	52.5
Hip extension							-0.04	-0.05	0.12	0.33	-34.5	-47.9	22.0	61.0
Hip flexion							-0.21	-0.19	0.12	0.34	-22.9	-31.1	15.1	42.0
**Secondary parameters Force (N)**														
Dorsiflexion	13.4	9.3	25.7	48.1	25.9	71.9	-12.8	-14.3	10.6	29.5	-20.8	-24.7	22.2	61.7
Plantarflexion	40.5	21.2	58.9	71.7	29.6	82.1	-4.4	-11.7	23.2	64.3	-6.5	-16.3	22.4	62.1
Knee extension	11.5	11.0	30.4	22.7	18.4	51.1	-7.0	-21.9	13.6	37.8	-14.4	-34.0	17.2	47.5
Knee flexion	29.7	22.2	61.6	68.7	31.8	88.3	-1.0	-6.0	9.4	26.0	-3.2	-14.3	13.6	37.6
Hip abduction							-11.9	-10.6	12.0	33.2	-24.2	-20.6	18.0	50.0
Hip extension							-4.4	-5.1	15.3	42.4	-28.3	-41.1	15.6	43.3
Hip flexion							-17.0	-21.2	16.9	46.9	-25.6	-21.6	12.6	34.8

Abbreviations in alphabetic order: A. Diff. = absolute difference; CP = cerebral palsy, DMD = Duchenne muscular dystrophy, MDC = minimal detectable change, N = newton, Nm = newton meters, Nm/kg = newton meters per kilogram; R. Diff. = relative difference, SEM = standard error of measurement.

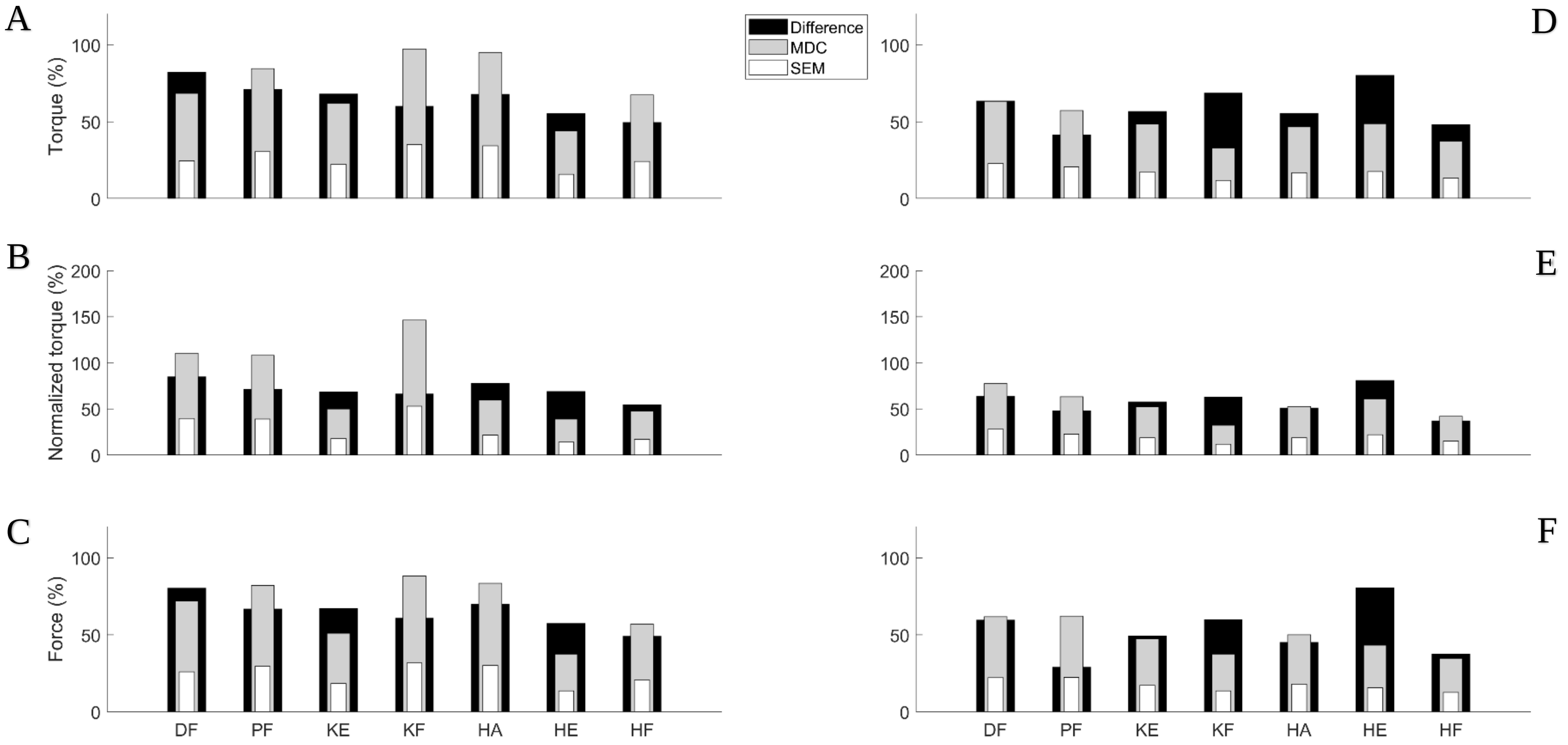

Supplementary figure 1: Visual representation to compare the relative SEM (white) and MDC (grey) values as retrieved from the reliability study for the children with CP and DMD with the relative differences between the median values of TD children and the clinical cohorts (i.e., the absolute difference between the median data relative to the median TD scores) (black) from the validity study. Panel **A-C** visualize the data for torque **(A),** normalized torque **(B)** and the force **(C)** in the children with CP. Panel **D-F** visualize the data for the torque **(D),** normalized torque **(E)** and the force **(F)** in the children with DMD. Abbreviations in alphabetical order: CP = cerebral palsy, DF = dorsiflexion, DMD = Duchenne muscular dystrophy, HA = hip abduction, HE = hip extension, HF = hip flexion, KE = knee extension, KF = knee flexion, MDC = minimal detectable change, N = newton, Nm = newton meters, Nm/kg = newton meters per kilogram bodyweight, PF = plantarflexion, SEM = standard error of measurement, TD = typically developing.

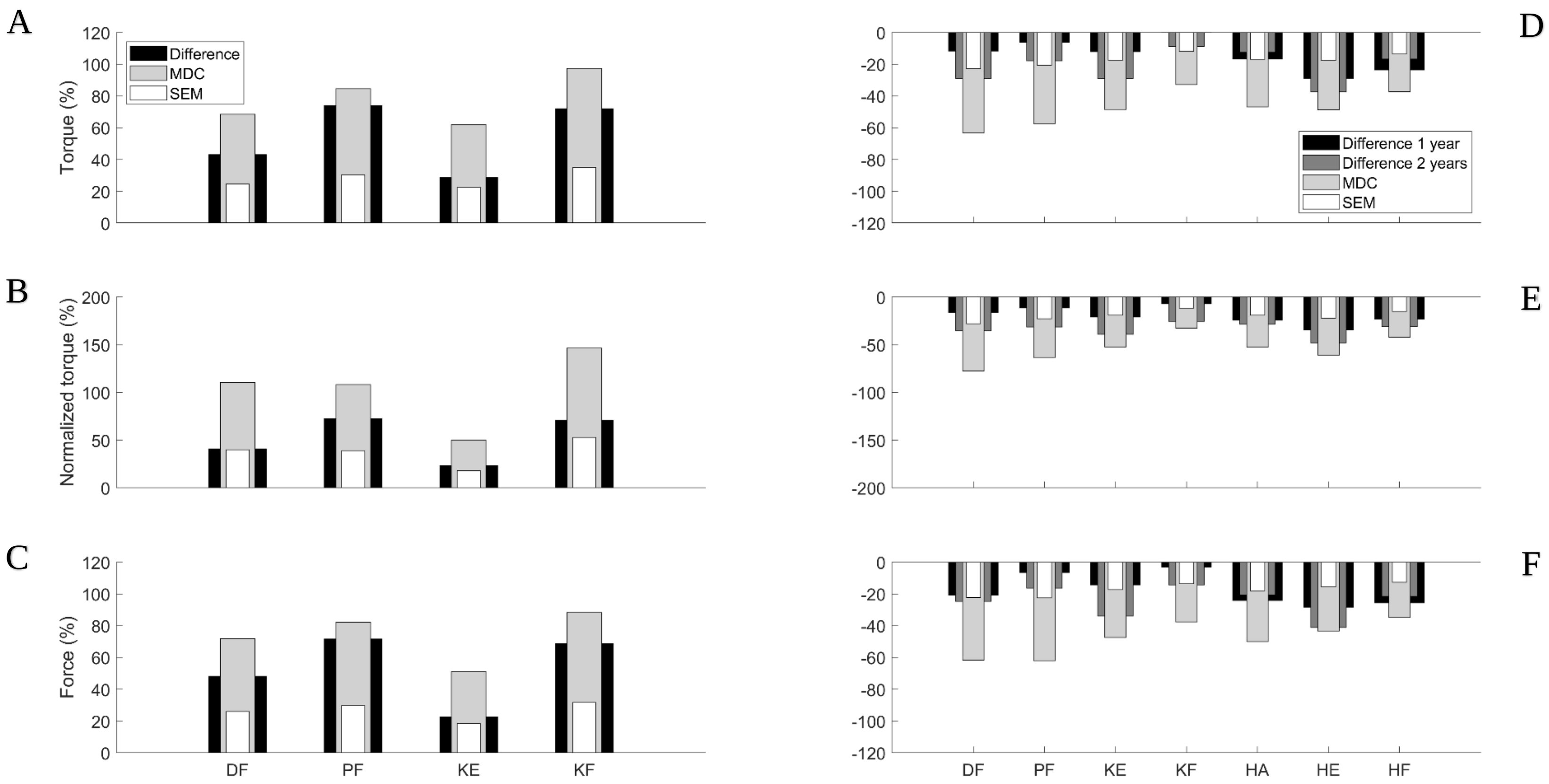

Supplementary figure 2: Visual representation to compare the relative SEM (white) and MDC (light grey) values as retrieved from the reliability study for the children with CP and DMD with the median of the relative differences (relative difference calculated as absolute difference between two assessments relative to the first assessment per participant) between the two assessments (black=CP and one year interval DMD; dark grey= two year interval DMD) from the responsiveness study. Panel **A-C** visualize the data for torque **(A)**, normalized torque **(B)** and the force **(C)** in the children with CP. Panel **D-F** visualize the data for torque **(D)**, normalized torque **(E)** and the force **(F)** in the children with DMD. Abbreviations in alphabetical order: CP = cerebral palsy, DF = dorsiflexion, DMD = Duchenne muscular dystrophy, HA = hip abduction, HE = hip extension, HF = hip flexion, KE = knee extension, KF = knee flexion, MDC = minimal detectable change, N = newton, Nm = newton meters, Nm/kg = newton meters per kilogram bodyweight, PF = plantarflexion, SEM = standard error of measurement.
